# Agent‐Based Modelling of Microbialite Formation Through Sedimentation and Precipitation Dynamics

**DOI:** 10.1111/gbi.70071

**Published:** 2026-11-04

**Authors:** Niall Rodgers, Laurane Fogret, Mark van Zuilen, H. James Cleaves, Sudha Rajamani, Sean McMahon

**Affiliations:** ^1^ UK Centre for Astrobiology, School of Physics and Astronomy University of Edinburgh Edinburgh UK; ^2^ Edinburgh Centre for Planetary Sciences University of Edinburgh Edinburgh UK; ^3^ Naturalis Biodiversity Center Leiden the Netherlands; ^4^ Department of Chemistry Howard University Washington DC USA; ^5^ Earth and Planets Laboratory Carnegie Institution for Science Washington DC USA; ^6^ Blue Marble Space Institute for Science Seattle Washington USA; ^7^ Department of Biology IISER Pune Pune Maharashtra India; ^8^ School of Geosciences University of Edinburgh Edinburgh UK

## Abstract

Fossil microbialites resulting from the interplay of sedimentation and precipitation are among the oldest evidence of life on Earth and help geobiologists to interpret many sedimentary environments. However, the factors governing their internal structure and external morphology are still poorly understood. Additionally, abiotic processes can mimic the morphology of some microbialites, complicating their use as ‘biosignatures’. The presence and style of internal structures such as clots and laminations may help solve both problems, but existing models of microbialite growth address only external morphology and do not show explicitly how internal structures emerge. In this work, we present an agent‐based model of microbialite formation using a fast, open‐source, high‐level microbialite modelling framework, written in Julia. We separate and label the products of sedimentation and precipitation, allowing external periodic forcing to control either or both. We qualitatively reproduce a wide range of internal and external morphologies found in real microbialites. Importantly, we find that microbialites with the same external morphology can differ greatly in their internal structure, a result impossible to observe with previous approaches. Further exploration and extension of our model may offer new approaches to resolving the ambiguity of potential microbialites in the rock record. Finally, we present the first interactive microbialite simulation to enable live parameter updating through an accessible interface that can be used as a tool for both research and teaching.

## 1 Introduction

Microbialites are organosedimentary structures resulting from interactions between microbial communities and their environment, including sedimentary dynamics and mineral precipitation processes (Burne and Moore 1987; Riding and Awramik 2000). They encompass a wide spectrum of morphologies—from planar to domical or columnar—and of internal architectures, commonly classified into stromatolites (laminated), dendrolites (shrublike), thrombolites (clotted) and leiolites (aphanitic or fine‐grained) (Grey and Awramik 2020).

Understanding microbialites is vital for reconstructing Earth's history, as they provide some of the oldest and most abundant evidence for early life on our planet (Van Kranendonk et al. 2008; Hickman‐Lewis et al. 2018). These structures can form in a wide variety of environments, including shallow marine, lacustrine and hydrothermal settings, and are found throughout almost all geological time, from billions of years ago to the present day (Fogret et al. 2024). Their formation reflects a complex interplay of intrinsic biotic factors and extrinsic environmental controls at the time of deposition (Reid et al. 2024). They may also be subject to post‐depositional alteration, such as diagenesis and metamorphism. For all these reasons, interpreting the final microbialite morphology and internal fabric is often difficult, and assessing the biogenicity of some candidate fossils remains a significant challenge (García Ruiz et al. 2002; Van Kranendonk et al. 2025). This ambiguity complicates our understanding of the evolution of the Earth's biosphere (as shown by the recent controversy over 3.7 billion year old purported stromatolites in Greenland (Nutman et al. 2016)) and has implications for the search for life beyond Earth (Ruff and Farmer 2016).

One way to tackle the ambiguity of morphology is to gain a better understanding of the generative processes leading to given structures and thus to assess the role of biology in their formation. Many approaches to this in the literature have focused on qualitative and conceptual models (Dupraz et al. 2013; Reid et al. 2024); however, computational models offer an alternative framework to explore microbialite growth in a way that allows hypotheses about the emergence of particular structures to be tested.

Despite this potential, work in this area has been sporadic and real application limited. Continuum models have been proposed that are too general to capture details of the formation process, are not widely used in the microbialite community, and can lead to contradictory conclusions regarding biogenicity (Batchelor et al. 2000; Batchelor et al. 2004; Batchelor et al. 2005; Cuerno et al. 2012). By contrast, agent‐based models can provide more localised insights into the interacting processes that lead to the formation of microbialites. The seminal work by Dupraz et al. (2006) introduced an agent‐based model to simulate microbialite growth, which has been widely cited by the community and recognised for its potential. However, it has not been widely applied, and relies on two‐decade‐old C++ code currently available only on request. In relation to stromatolites, both approaches suffer the same fundamental limitation, which is that visible laminations do not arise from interactions within the models. Although illustrations of these models often appear to show lamination, these are drawn artificially and represent former positions of the external surface during growth. The absence of true internal structure limits the applicability of these models to the whole spectrum of microbialites. The same point was raised by Dupraz et al. (2006) themselves: ‘the model does not produce a mechanistic description of the laminae but rather a holistic approach to morphology generation’. This has thus far remained unaddressed.

To resolve this, we here present a fast high‐level agent‐based model of microbialite formation, developed in Julia using the Agents.jl framework. Our model builds upon the approach of Dupraz et al. (2006) but introduces key enhancements, not present in the original work: (i) it explicitly labels sedimentation and precipitation as separate processes, (ii) it allows external periodic forcing to influence layer formation, (iii) it is available publicly and written in a high‐level extensible framework and (iv) it can be run interactively allowing live parameter updates. The model simulates these processes using a framework inspired by diffusion‐limited aggregation (DLA), allowing both mechanisms to be individually labelled and tracked. This makes it possible to explore how both macroscopic morphologies and internal fabrics can emerge from the interactions, and stand‐alone behaviour, of precipitation and sedimentation.

Firstly, we show how this model can reproduce a wide range of microbialite varieties. We then qualitatively explore the morphospace generated by the model and show how an interactive interface can be used to easily approximate structures containing multiple sequential morphological styles. Finally, we demonstrate how the model reveals the decoupling of internal patterning from external morphology, highlighting the complexities of microbialite formation. In the Supplementary Information, we highlight how the model can be modified to heuristically generate conical‐like objects, subject to limitations, and how various quantitative measurements can be used to understand the model output. Although this high‐level model represents an exploratory framework of microbialite formation and is still limited in realism and scope, it provides a flexible and extensible foundation for future microbialite modelling. Moreover, the model directly demonstrates the ambiguity of morphology as a biosignature, since by using simple abstract rules and external periodicity a wide spectrum of morphologies can be generated without any reference to the underlying details of the true mechanisms or their biogenicity.

## 2 Previous Approaches to Microbialite Modelling

There is a long history of the computational study of microbialites (primarily focused on stromatolites) with simple computer programs implemented by Hofmann (1969) over 50 years ago. However, the work has been sporadic and no standard approach has emerged (Bosak et al. 2013). The state of the numerical modelling described in the decade‐old review of Bosak et al. (2013) remains essentially unchanged. Broadly, two main modelling approaches have been pursued. The first involves representing stromatolite growth through partial differential equations, capturing surface evolution (Grotzinger and Rothman 1996; Batchelor et al. 2000; Cuerno et al. 2012; Petroff et al. 2013; Ojakangas et al. 2023). The second focuses on stochastic agent‐based (or process‐based) models, which aim to simulate the localised, dynamic interactions between sedimentation and microbial processes (Dupraz et al. 2006; Johnson and Grotzinger 2006; Curtis et al. 2021).

### 2.1 Continuum Models

The continuum modelling of microbialites began with the work of Grotzinger and Rothman (1996) who proposed that stromatolites could be modelled by the Kardar–Parisi–Zhang (KPZ) equation, a framework originally developed to describe generic surface growth, where they directly aimed to answer questions of biogenicity. This marked a shift toward viewing stromatolite morphogenesis as a physical process rather than just a biological one. Further work extended and analysed the applicability of KPZ to stromatolites and biogenicity (Batchelor et al. 2000; Batchelor et al. 2004; Batchelor et al. 2005; Cuerno et al. 2012; Ojakangas et al. 2023).

Despite their foundational role in showing the ambiguity of morphology as an indicator of biogenicity (Grotzinger and Rothman 1996), KPZ‐based models have significant limitations when used to model microbialites, discussed in detail in Cuerno et al. (2012). Some types of stromatolite morphologies, in particular overhangs, cannot be generated by KPZ models. As a highly general description of interface growth, the KPZ equation offers a limited capacity to distinguish between biotic and abiotic processes, and its terms cannot be directly tied to specific mechanisms of microbialite formation. Additional caveats regarding asymptotic limits are given in Cuerno et al. (2012).

An under‐emphasised point is that when using KPZ models the layers must be drawn artificially; each layer represents the height of the structure at a particular time, rather than sedimentary or compositional variation of the kind found in real stromatolites in the rock record. The models also only allow an understanding of gross external morphology and cannot be used for exploring transitions between different microbialite types. Additionally, KPZ models are not well suited to the microbialite community as it is difficult for non‐mathematical specialists to interpret the equations. Furthermore, it is difficult to test specific hypotheses surrounding biogenicity or formation, although this can be attempted as in Ojakangas et al. (2023). For all these reasons, since the initial peak of the 1990s and early 2000s we have seen limited uptake of this approach to modelling stromatolites.

An interesting physics‐based continuum approach is found in the work of Petroff et al. (2013), who argue that the shape of conical stromatolites derives from the physics of diffusion through a thin film. Although this promising approach can explain the external morphology of conical stromatolites and their laminae, it does not explain why lamination occurs. In addition, it behaves in similar ways to the models of the formation of abiotic conical structures like stalactites and icicles. Abiotic objects of this specific class can be well represented by other continuum models, such as those developed by Szymczak et al. (2025), yet this cannot be easily achieved for generic microbialites due to the complexity of interacting processes and the diversity of resulting types in the fossil record.

Although continuum models are an efficient (if potentially mathematically complex) way to capture broad morphological trends, they often struggle to represent internal structure. In many cases, they are too generic to test real‐world hypotheses or draw strong conclusions about biogenicity.

### 2.2 Agent‐Based Approach

An alternative approach to modelling the formation of microbialites is to use an agent‐based model (ABM), where large numbers of agents interacting according to local rules combine to generate emergent morphologies. This approach is exemplified by the seminal work of Dupraz et al. (2006) who combined a diffusion‐limited aggregation (DLA)‐like model of sediment with a cellular automata model of precipitation to reproduce a range of microbialitic morphologies. While this model has been widely cited, its direct real‐world application or extension in the literature has been limited. The original model reproduces the external morphology of stromatolites but not their internal laminations (unless these are drawn artificially by recording the history of the growing surface). As far as we are aware, no peer‐reviewed article has yet proposed a formal extension of the model. Additionally, while this model follows the reproducibly standards of its time (nearly two decades ago), it is written in C++ and the source code is only available on request.

Agent‐based models (ABMs) have great potential benefit to the study of microbialites, whose morphologies emerge from local interactions. A variety of biological and environmental processes could be represented by interpretable local rules, enabling targeted hypothesis testing and access to internal structural dynamics. In 2006, Johnson and Grotzinger (2006) offered a glimpse of this potential when they used a process‐based numerical model to relate the structural features of stromatolites in Namibia to the sedimentary regime in which they formed; however, as the authors state, ‘The model cannot explain detailed morphologic attributes’. Metazoan–microbial reef growth was also simulated in the simple 3D numerical model of Curtis et al. (2021), which again does not capture the internal structure.

A parallel line of research focuses on the modelling of branching abiotic mineral dendrites, again taking inspiration from DLA processes (Chopard et al. 1991; Hou et al. 2025). Recent work using Lattice Boltzmann methods to simulate the formation of these structures (Hou et al. 2023) reproduces gross morphologies somewhat similar to those produced by our model in the branched growth regime. This highlights the potential convergences of biotic and abiotic processes.

## 3 Proposed Model

### 3.1 Goals of the Model

The goals of the model presented are to capture the range of morphologies present across the spectrum of microbialites (and potentially abiotic morphologies) within a generic, parsimonious framework. This model is intended as a starting point for the resumption of agent‐based modelling of microbialite formation, leaving scope for extensibility or specialisation.

We do not claim or attempt to capture the full range of physics, biology and chemistry underlying the multitude of interacting processes that affect the formation and preservation of microbialites. However, our model is intended as a conceptual and exploratory tool, providing a step forward compared to previous work and building on technological developments that make it worthwhile to return to this topic.

The core assumption of the model is that internal structure arises from the interplay of sediment trapping and binding, precipitation, as well as periodicity in both of these phenomena. Additional features, types and causes of laminations and internal morphologies result from the complex interplay of biotic and abiotic processes, which would require an updated framework to capture. Examples include preserved organic matter (Knoll and Semikhatov 1998; Allwood et al. 2009), mineralogical variation (Djokic et al. 2017; Baumgartner et al. 2024), variations in detrital grain size (Suarez‐Gonzalez et al. 2014), syn‐depositional deformation and fluid‐filled fenestrae (Sumner 2000; Rivera and Sumner 2014).

The model necessarily contains a large number of parameters; we detail these in the Supplementary Information. Although there are relatively few controlling parameters, there are many generic computational parameters (21 in total). Some are simple Booleans while others could be replaced by any appropriate mathematical function. For practical reasons discussed in the Supplementary Information we avoid parameter non‐dimensionalisation.

### 3.2 Modelling Framework

In this work, we use the Agents.jl (Datseris et al. 2024) agent‐based open source modelling framework. We choose this approach for several reasons. First, Julia is an accessible high‐performance programming language. Second, such a framework allows us to focus on the dynamic rules implemented rather than the details of the ABM implementation, which are handled and optimised by the framework. Third, the choice of Julia and Agents.jl aids extensibility as all the underlying code is pure Julia, simplifying the process of modifying the model with fast user‐friendly code. Finally, this framework supports parallel computing; parallel simulations can be initialised in a few lines on a laptop, making it possible to explore large morphological parameter spaces. Additionally, the framework can be used to run the model interactively, allowing live parameter exploration.

### 3.3 Setup of the Model

The model simulates the formation of a two‐dimensional microbialite, drawing inspiration from the model of Dupraz et al. (2006), using a grid with periodic horizontal boundary conditions. Extensions to three dimensions could be considered in future work (and would be simplified by the Agents.jl framework). Our model, like that of Dupraz et al. (2006), consists of two separate processes, each represented by a distinct set of agents. One group of agents represents sediment particles that are trapped and bound by ‘microbial activity’ (or generic abiotic adhesion) on the surface of a solid substrate. The other group comprises precipitating agents that reside at the solid–fluid interface and represent an active microbial layer that can induce mineral precipitation or abiotic surface precipitation. We do not attempt to simulate the detailed dynamics of the biofilm or abiotic dynamics; instead, we focus on the events of precipitation and sedimentation (trapping and binding), as these dominate the features most likely to be preserved in the geological record and observed in fossil microbialites.

Each agent or grid cell does not represent an individual sediment grain, microbe or abiotic precipitated grain but instead the aggregate effects of a collection of processes at a larger scale. Each agent type can fill cells of the grid, leading to growth in the solid structure. When each agent type fills a cell this is marked with a distinct value, allowing for clear differentiation between precipitated and sediment‐derived material when visualised as colours. The different values can be taken to represent some physical property (e.g., grain size) or chemical characteristics (e.g., mineralogy or elemental composition) that would allow sediment and precipitate to be differentiated visually or via petrographic techniques or elemental mapping such as energy‐dispersive X‐ray spectroscopy (EDX) (Hickman‐Lewis et al. 2023). In this work, we plot precipitated mineral particles in yellow and trapped/bound sediment grains in red. Although separate agent classes were a feature of the work of Dupraz et al. (2006), our model is the first to use separate labelling of agent classes and their products to generate internal morphologies.

Distances between cells in the model are measured using the Euclidean distance function in Agents.jl. Agents update (with one agent per cell) in a random order to reduce scheduling artefacts when interacting with the structure, which may change during their dynamics. The average time for a particle from release to instantaneous stick may also change as structure grows in a non‐uniform manner.

### 3.4 Sediment Agents

The sediment dynamics of the model represent a high‐level approximation and aggregation of real‐world physics. A random walk approximates the settling dynamics of the sediment through the water with basic shadowing dynamics emerging from the column tips. The stability and attraction distances provide an approximation of the adhesion and settling of the agents onto the surface which would represent an aggregation of the adhesive properties of the surface (related to EPS production when assuming a microbial mat), how the agent settles (related to hydrodynamics and adhesion) and the ability of the structure to maintain dendrites or columns. The model parameters can be interpreted in different ways and do not necessarily require a biological gloss. However, on some interpretations it may be possible to use the model to explore how biological and non‐biological sedimentary structures differ, since the dynamics of abiotic sediment settling and sediment adhering to a microbial mat give different levels of structural support and allow different geometries to emerge (Tice et al. 2011; Frantz et al. 2015).

The dynamics of sediment agents draw from the work of Dupraz et al. (2006) and hence the physics of diffusion‐limited aggregation. We use the term DLA‐like to acknowledge the literature terminology associated with stromatolite modelling (Dupraz et al. 2006) while noting this differs from the physics usage. We note that the way DLA is used in the physics literature may imply a Laplace field, whereas our model uses a Poisson field, with particles starting moderately offset from the structure, undergoing a biased random walk with random update scheduling. The term DLA‐like was retained to invoke the random walk and emergent dendritic morphologies which arise from this process while also matching the literature convention (Dupraz et al. 2006). The moderate offset is required for computational efficiency.

At each time‐step, a given number of sediment agents are introduced to the simulation. These agents are given two agent‐level properties that impact the morphology: the stability distance and the attraction distance. A sediment agent that has not yet attached to the solid structure will look for locations directly adjacent to solid filled cells within its attraction distance radius from which to conduct a stability search. The agent then sequentially selects the locations from the randomly shuffled list within the attraction distance for a stability distance search until successful (see schematic, Figure 1).

**FIGURE 1:**
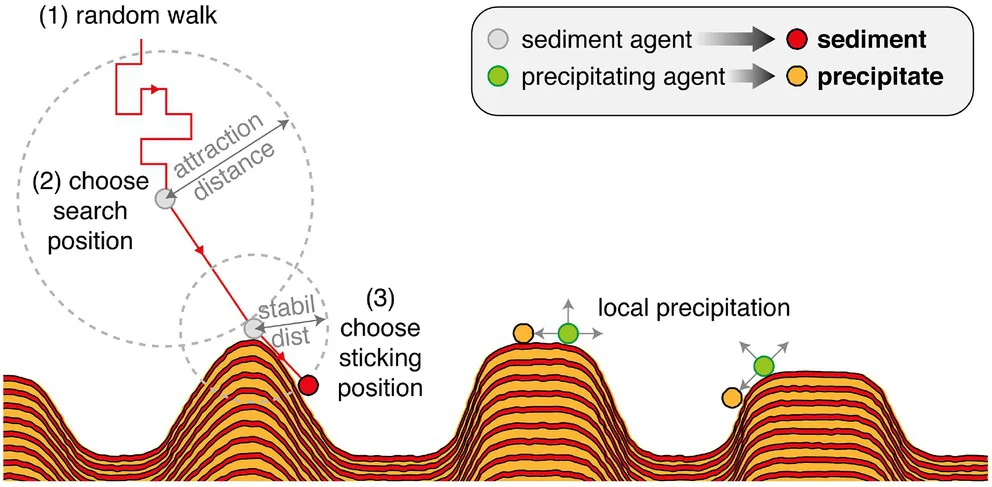
Schematic overview of the model showing the dynamics of each agent type. Figure inspired by Dupraz et al. (2006).

During the stability search within its stability distance, the agent looks for a valid and preferred location to ‘stick’ and fill a cell with labelled solid. A location is valid if it is currently empty and has at least one neighbouring solid‐filled cell. From valid locations, the sticking site is selected stochastically. The probability of sticking at site *i* is given by:

(1) $$p_{i}^{\text{stick}}=\frac{\nu_{i}}{\sum_{j}\nu_{j}},$$

where the sum of cells in the denominator runs over all valid locations, and $\nu_{i}$ is the number of solid neighbours for a given candidate cell.

This formulation gives preference to sites that are more structurally stable, adjacent to more solid material. If the agent sticks successfully, the grid cell is marked as solid sediment, and the agent is removed from the simulation. With a given recolonisation probability, the site of the newly stuck agent can acquire a precipitating agent, representing microbial or abiotic activity, which can initiate local mineral precipitation. If no places to stick are found, the agent undergoes a biased random walk with equal probability of moving left or right at a given horizontal speed and a biased probability (set to 90% in this study) of moving down rather than up at a given vertical speed, to force downwards motion with a small amount of randomness. The walk function uses fixed step sizes, and the agent cannot walk into other agents, as we enforce one agent per cell. If a sediment agent hits the bottom of the grid or walks into the solid, it is removed from the simulation. The walk function could be modified to introduce directional bias (e.g., simulating flow or current without full hydrodynamic simulation) or made time‐dependent to simulate environmental conditions.

### 3.5 Precipitating Agents

The precipitation agents in this model represent generic mineral precipitation which may be microbially induced or abiotic in origin. Differentiation of abiotic and biotic processes may be difficult in practice (Bosak et al. 2013) and many relevant objects may form as a result of abiotic precipitation (Cantine et al. 2020; Riding 2008). The probability of precipitation, and potential directional bias, represents the aggregation of the environmental factors which drive precipitation: temperature, pH, chemical concentrations, for examples, concentration of dissolved ions and potential microbial activity. Because many of these properties can also vary daily, seasonally or on larger timescales, we allow for periodic variation in precipitation rates. Precipitation agents that were interpreted as representative of microbial activity were a feature of the study by Dupraz et al. (2006). However, they used a different, less expressive cellular automata scheme, which does not allow as strong control over the precipitation dynamics and periodicity.

Precipitating agents can be introduced either by the growth of another precipitating agent, by recolonisation triggered by a sediment agent sticking, or by the setup of the initial conditions. At the start of each step, precipitating agents may be removed with a given probability to simulate random processes that could interfere with agents (e.g., grazing, metabolic shutdown or erosion). If the agent remains, it loops over each of its immediate neighbouring cells (in a given metric, which includes diagonals for the Euclidean case), selects the empty cells, and assigns each of these cells a weight, $w_{i}$, indexed from a parameter array, depending on whether it is up, down or horizontal relative to the cell. A cell is then selected for potential growth with probability

(2) $$p_{i}^{\text{grow}}=\frac{w_{i}}{\sum_{j}w_{j}},$$

where the denominator of the fraction is a sum over all valid growth directions. Growth to the selected cell then occurs stochastically according to the current value of the precipitation probability. When an agent grows, it fills the selected cell with labelled solid and creates a precipitating agent at that location. Finally, precipitating agents that become completely surrounded by solid‐filled cells are removed from the simulation, mimicking the smothering of microbial mats or of favourable nucleation sites for abiotic precipitation.

### 3.6 Initial Setup

We use Agents.jl to initialise a model and select a random scheduler to prevent sequencing artefacts. The model starts with an initial solid substrate, labelled distinctly from either sediment or precipitate. This initial substrate can be of any shape. However, we limit it to five configurations in this work: a single flat horizontal layer, a localised horizontal patch, a small mound, a small triangle or sinusoidal fluctuations all along the base of the model. Onto this solid substrate, we place precipitating agents with a probability according to the defined initial density as detailed in the Supplementary Information.

### 3.7 Periodicity and Sediment Addition

In order to grow the structure, we introduce sedimenting agents over time as they are continually depleted when forming the solid. In previous work (Dupraz et al. 2006), sedimentation was generally introduced at a constant rate. However, this does not need to be the case, as periodic sedimentation can be incorporated into the model. Moreover, independently of any periodicity in the sedimentation, there may be externally forced periodicity in the rate of precipitation (microbially induced or otherwise). The varying strength of either of these sources of periodicity, or the interaction between them both, may parsimoniously explain the emergence of the varied internal structures of microbialites. In physical terms, periodicity may occur in microbial activity, carbonate or counter‐ion concentrations, background sedimentation rate or almost any other environmental process likely to modify the rates of precipitation or trapping and binding on any appropriate time scale (daily, weekly, seasonally, etc.).

In our model, to minimise as much as possible the number of parameters, we modulate the precipitation rate and the sedimentation rate in the following way. We set the sedimentation rate equal to

(3) $$\text{sedimentation}\;\text{rate}=\text{round}\left(A_{\text{sediment}}f_{s}\left(\frac{2\mathrm{\pi t}}{T}\right)\right),$$

where $A_{\text{sediment}}$ is the maximum sedimentation rate or sediment amplitude and T is defined as the period control parameter. We also round the sedimentation rate as we cannot add a non‐integer amount of sediment. The modulation $f\left(\frac{2\mathrm{\pi t}}{T}\right)$ can be any function that takes values $\left[0,1\right]$ and depends on the number of time‐steps passed, t. In order to reduce the number of parameters, in this study we couple the periodic modulation of the precipitation rate to the modulation of the sedimentation rate (although they are logically distinct and could be decoupled in future work). The precipitation rate is defined as

(4) $$\text{Precipitation}\;\text{probability}=A_{\text{precipitation}}f_{p}\left(\frac{2\pi \left(t+b_{\text{offset}}\right)}{a_{\text{scale}}T}\right),$$

where repeated parameters are as in the sedimentation rate definition (Equation 3), $A_{\text{precipitation}}$ is the maximum precipitation rate or precipitation amplitude, which is a probability $\left[0,1\right]$. While $b_{\text{offset}}$ and $a_{\text{scale}}$ set the way two mechanisms interact.

We allow independent functions, $f_{p}$ and $f_{s}$, for the precipitation rate or the sediment addition rate to be chosen from the following options in addition to constant behaviour: $\max\left(\cos x,0\right)$, $\cos^{2}x$ or ∣cosx∣. Hence, we refer to T as a ‘period control parameter’ as when changing functions and scaling etc. It may no longer be the true period but will simply act as a related control parameter. Due to the generality of the model, any parameter can be made time‐dependent and updated according to any function (periodic or not), which respects the constraints of the model dynamics. For simplicity, in this work we largely set the periodic function to $\max\left(\cos x,0\right)$, as it most clearly promotes layer formation. However, we find that lamination can still emerge using other functional forms.

For each given sedimentation rate, we add sediment agents into the grid by the following process. Rather than releasing all agents at the top of the grid, we release from a lower height for computational efficiency, with the addition of some randomness to prevent computational artefacts. Then, we select a set of possible input locations by several steps. Firstly, we determine the base input row as the minimum between the height of the grid minus 5 and the maximum height of the solid plus 10 times the vertical step size. A random downward offset between 1 and the step size minus 1, is applied to this base row. Secondly, using this base row, we select all valid locations not already solid or containing an agent across all columns and ±5 rows in either direction. Finally, from this set of possible locations, we randomly draw (without replacement) until we have added the required number of sediment agents.

### 3.8 Overview of Model

This model separates sedimentation (which could be interpreted as microbial trapping and binding) from (potentially microbially induced) precipitation, giving insight into emergent morphology and allowing hypotheses to be tested. Due to the nature of agent‐based modelling, there are a large number of parameters and some choices that need to be made by the modeller, which are arbitrary within the realm of reasonableness, such as how exactly to select the release height positions. This model is also not necessarily unique in that there are likely other ways to simulate the microbialite formation process. However, this work provides a principled advance over previous approaches (Dupraz et al. 2006).

It is important to note that the current model works only in simulation units, rather than being connected directly to physical time or space. Linking a model of this kind to physical units may be the subject of future work. However, it is difficult to precisely calibrate against the growth rates of real world microbialites, while aspects of the morphological patterns generated by DLA are scale‐invariant by nature (Witten and Sander 1981). When running the model, we impose a maximum number of time steps or, when producing visualisation and morphospace plots, stop the run when the maximum height of the structure reaches within 50 cells of the top of the domain.

## 4 Results

### 4.1 Comparison to Real Microbialites

Microbialites exhibit a remarkable diversity of forms shaped by the interplay of environmental conditions and microbial processes over various timescales (Grey and Awramik 2020; Fogret et al. 2024). Although the full morphological spectrum cannot be fully captured in just a few figures, we present a curated selection of representative examples (Figure 2) from different geological periods, which highlight key structural characteristics observed in the field. These comparisons serve as qualitative targets for our simulations. We find that our model is capable of qualitatively reproducing a broad range of these morphologies under different parameter settings.

**FIGURE 2:**
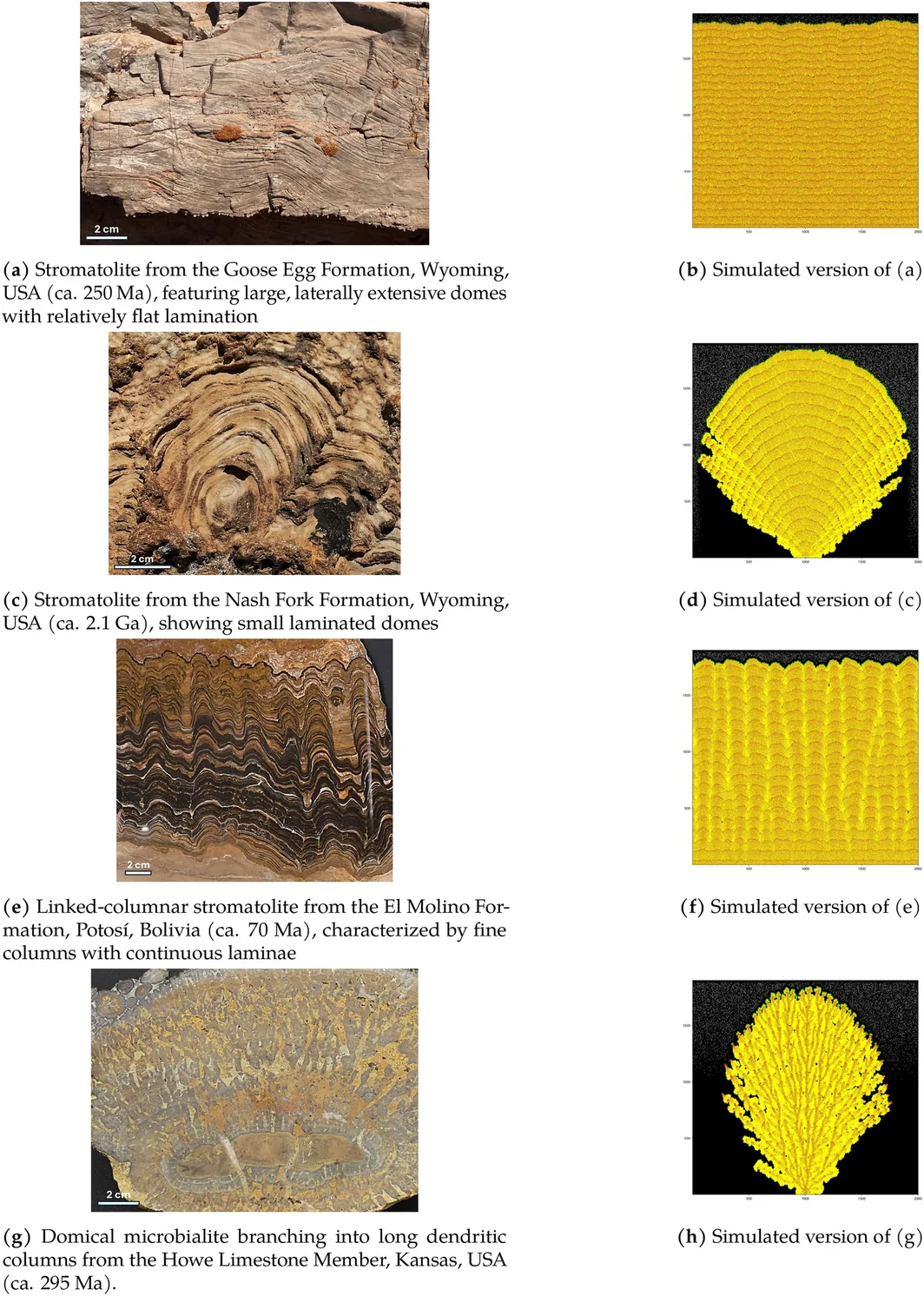
Comparison of real microbialite specimens (left column) and simulated analogues produced by our agent‐based model (right column), under distinct parameters. In simulation panels, precipitation is shown in yellow and sediment in red. All parameters listed in the Supplementary Information.

Our simulation does not aim to replicate these structures at the microscopic level or match precise growth mechanisms. However, our ability to approximate such diverse morphologies with a limited number of adjustable parameters demonstrates the generative and perhaps explanatory power of the model. This approach bridges the gap between field observations and computational modelling, offering a conceptual framework to explore how environmental and microbial controls may shape microbialite architecture across geological time.

In Figure 2, we show how our model qualitatively reproduces a range of microbialites with laminations arising from the periodic interplay between the agent types in the model. One caveat with this approach is that the real object is three‐dimensional whereas the images show two‐dimensional slices. This creates geometric artefacts that can modify the appearance of branches, which are difficult to replicate in a two‐dimensional model. Parameters of the reproductions are listed in the Supplementary Information.

### 4.2 Exploring the Parameter Morphospaces

One way to elucidate the behaviour of the model is to plot the generated structures into a morphospace. Morphospace analysis has been a standard way to understand the observed diversity of natural forms since the famous shell morphospace of Raup (1966) with many pattern‐forming systems, such as skin pigmentation in hybrid fish (Miyazawa et al. 2010), being amenable to this approach. By using our parameter sweeps to construct a morphospace, we show how a wide diversity of microbialite forms may be generated using comparatively few controlling parameters.

As there are many control parameters for this simulation, we cannot visualise them all simultaneously in one plot. However, in Figure 3 we show a subset of key parameters that most strongly influence the emergent external morphology.

**FIGURE 3:**
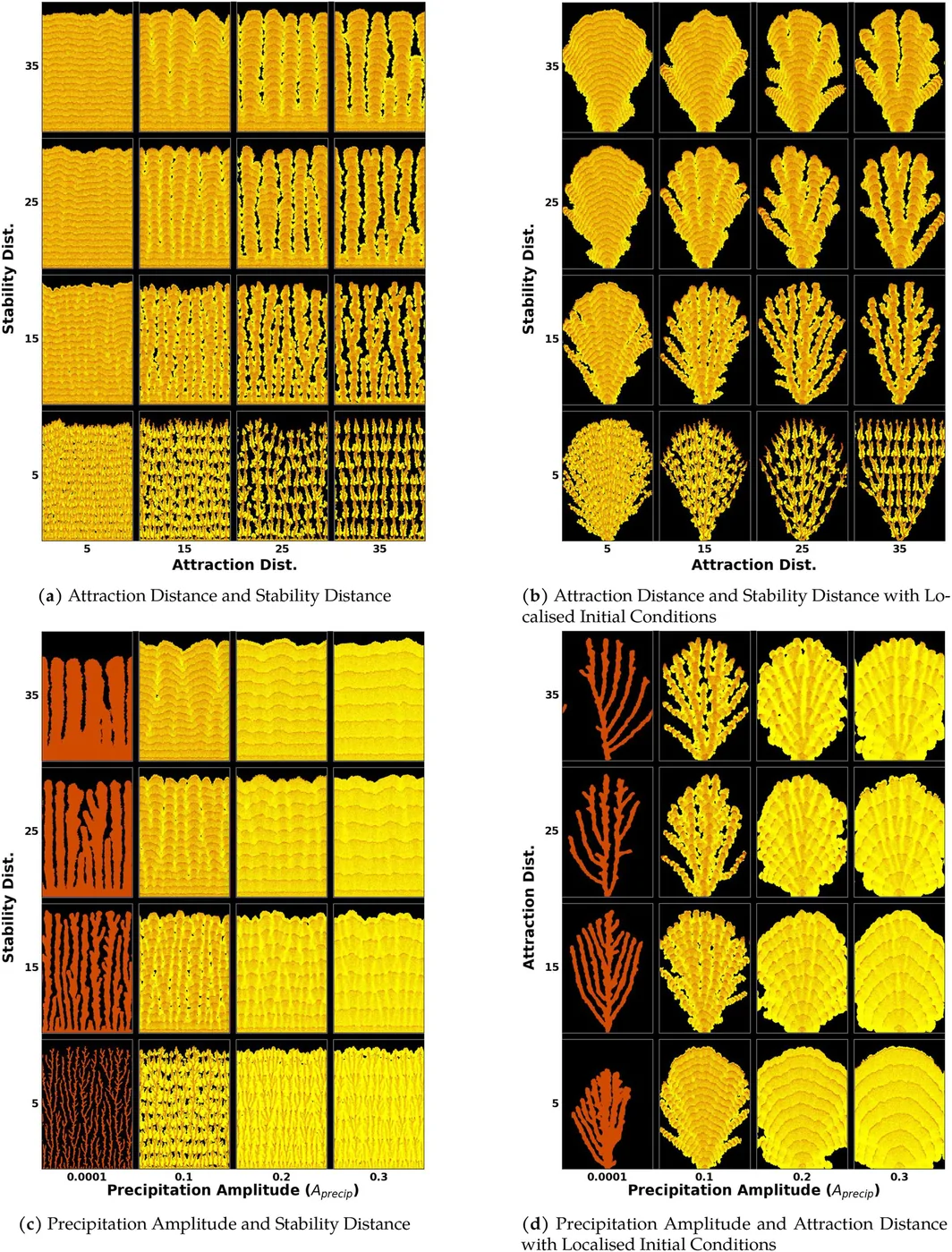
Parameter morphospaces. Demonstration of the wide range of possible morphologies generated and the parameter dependence. Precipitation in yellow, sediment in red. Parameters in Supplementary Information.

Specifically, we explore the effect of varying two of attraction distance, stability distance and precipitation amplitude at a time, while holding all other parameters constant (see Supplementary Information for values). We show simulations under both localised and uniform initial conditions to highlight how the initial setup interacts with model dynamics to shape the final structures.

The first morphospaces (Figure 3a,b) show how the stability distance and the attraction distance control the output morphology. High stability distance combined with low attraction distance leads to flatter layered structures. High stability distance combined with high attraction distance leads to columnar structures. Low stability distance combined with high attraction distance leads to sparse branching structures. Low stability combined with low attraction distance leads to smaller‐scale branching, which is partially overwhelmed by mineral precipitation in this example.

Our results mirror those of Dupraz et al. (2006) (Figure 3) where similar trends are observed, albeit at different scales. Figure 3b,d shows the impact of spatially localised initial conditions and how this directly leads to radial or domal morphologies as the structure grows from a central point. This is important because many real‐world microbialites have this morphology, which is parsimoniously explained by growth from a localised nucleus.

Figure 3c,d shows how changing the rate of microbially induced precipitation leads to variations in morphology. Very low precipitation rates lead to sedimentation (trapping and binding)–dominated DLA‐like (terminology used in the style of Dupraz et al. 2006) morphologies without layering. At sufficient growth rates, regular layers can appear, alternating between sediment‐ and precipitate‐dominated phases, with layer thickness depending on the periodicity and balance of the two parameters. Internal branching or clotted patterns can also be maintained as the structure grows. All of these reflect the possible diversity of internal structure of microbialites and demonstrate the interactions between external and internal processes.

### 4.3 Interactivity and Abrupt Morphological Transitions

The parameters that govern the formation of real microbialites may not be constant throughout the development of the structure, either gradually varying or suddenly changing. Any parameters in this model can be made time‐dependent by updating the values during each time step according to any function or rule. However, this leads to an explosion of possibilities that could be tested and structures that may emerge. In order to address this and additionally make the work more accessible, we include an interactive simulation using Agents.jl (Datseris et al. 2024) and the abmexploration function. This version allows the simulation to launch in an interactive window, where a user can watch as the simulation progresses, as well as update the values of selected parameters, live as the simulation runs, using sliders. This interface and an example structure are shown in Figure 4. This can be accessed by running the code included in the Supplementary Materials. In the supplementary material, we include several videos of this in progress. A guide on how to run and install the code is given in the Supplementary Information.

**FIGURE 4:**
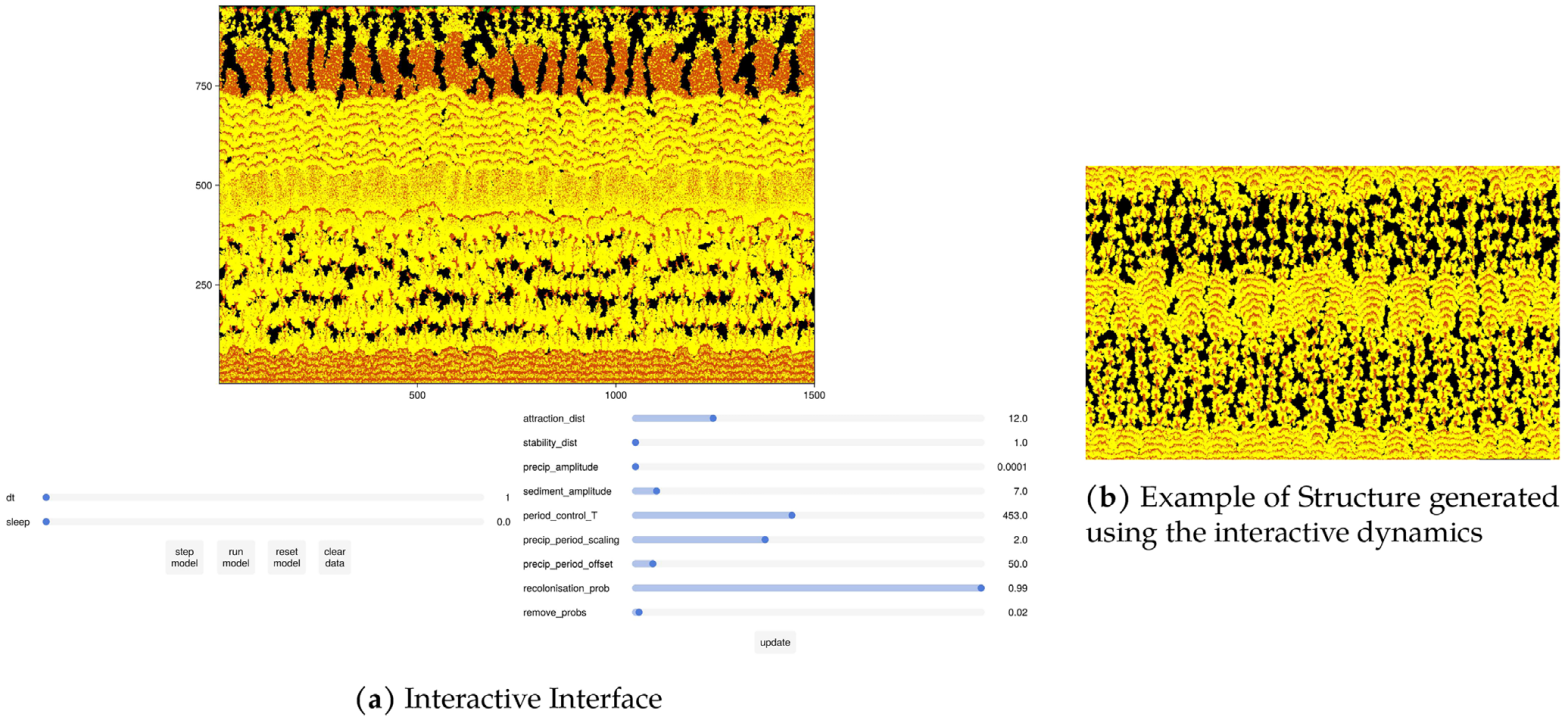
Interactive interface and morphological transitions. (a) Screenshot of the Interactive Interface showing sliders which allow parameters to be modified during the run and an example structure created by this process. (b) Simulated structure with sharp morphological changes generated by abrupt parameter changes using the interactive mode.

This feature allows insight into how parameter variations can lead to a whole range of structures, and could also be used as a teaching aid demonstrating theories of microbialite formation. Recognising this complexity is crucial when attempting to infer palaeoenvironmental conditions from fossil microbialites. Parameter variation during runs was shown in the work of Dupraz et al. (2006). However, our model includes internal fabrics and allows the user to see intuitively how they emerge from various parameters.

### 4.4 Decoupling Internal and External Morphologies

A key step forward in this work is the ability to generate a variety of internal structures and external morphologies as the simulated microbialite grows. This allows us to illustrate the complexity and contingency of microbialite formation and classification. Figure 5 illustrates how approximately similar external morphologies can occur despite radically different internal structures. This internal diversity results from different values of the period control parameter, which influences the emergence of stromatolitic, dendrolitic, leiolitic or thrombolitic internal fabrics.

**FIGURE 5:**
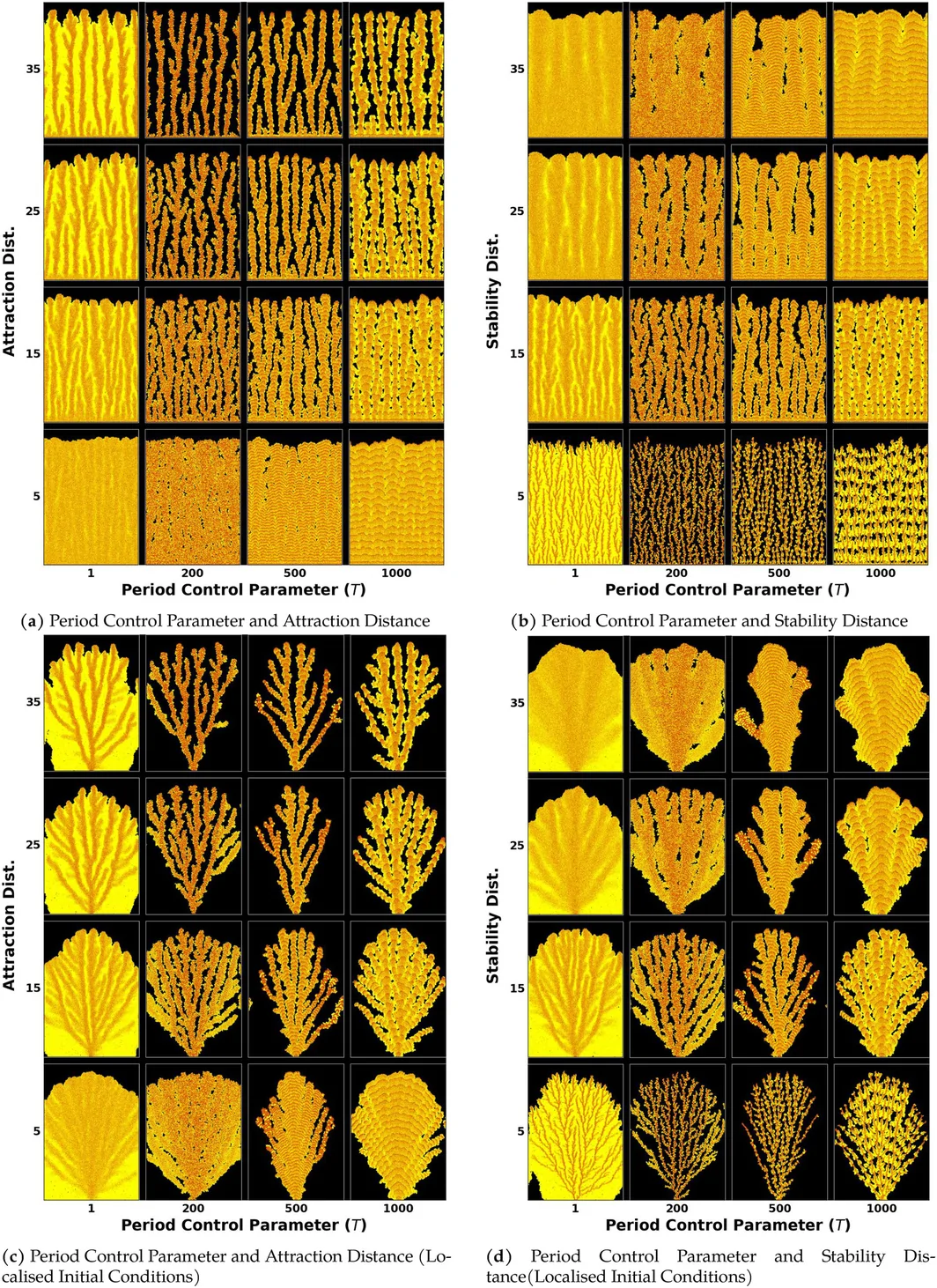
Internal fabric transitions: Variations in internal morphology with changes in external periodic control demonstrating transitions between microbialite types in several morphospaces.

For various values of stability and attraction distances, Figure 5, we plot the morphological varieties encountered when varying the periodicity control parameter. As we change the periodicity control parameter, we see a transition between discrete layers and the breakdown of lamination into random noise via clotting. This suggests that the magnitude of external periodic forcing governs a spectrum of internal fabrics from leiolites to thrombolites and stromatolites. Changing the external periodicity also modifies the rate at which material accumulates so that during near‐constant precipitation the intercolumnar spaces are filled with precipitate. This demonstration, Figure 5 suggests a degree of equifinality in microbialite form: different combinations of parameters can produce similar final forms, potentially limiting or complicating efforts to use microbialites as ‘environmental dipsticks’. The decoupling of internal and external morphologies suggests that the combination may give greater insight into the formation mechanism than one alone. Laminations give more information about the growth history of a microbialite than amorphous randomness, which may be more difficult to interpret. Additionally, in reality, a real object is only observed when dynamics have ceased, making it difficult to separate rate, speed and time uniquely depending on how laminations may or may not record the evolutionary history.

### 4.5 Additional Model Features

#### 4.5.1 Quantitative Measurements, Parameter Sweeps and Equifinality

Another useful aspect of this model is the ease with which the resulting structures can be characterised quantitatively, either aggregating or separating the two generative processes. A huge number of potential summary statistics could be used to describe the model outputs, including measurements inspired by the compression‐based complexity indices described in (Corsetti and Storrie‐Lombardi 2003; Wagstaff and Corsetti 2010). In the Supplementary Information, we give examples of some of these measures and show how they correlate with the input parameters and each other. Our framework makes it relatively simple to run independent simulations in parallel using high‐level Julia multithreading. This speeds up the sampling of the parameter space. However, there are still a large number of variables that could contribute. In order to simplify the process, we use Latin hypercube sampling, which allows the samples to be evenly distributed across parameter space while controlling the number of samples, and focus on the key parameters of stability distance, attraction distance and precipitation rate. One key conclusion of this analysis is the suppressive nature of certain parameters. Different strengths of correlations can be observed depending on the inclusion of precipitation rate in the sweep; if precipitation rate is too large, it can dominate the structure and hide other effects.

Quantitative analysis reveals two subtly different types of equifinality. Firstly, parameter‐dominance equifinality occurs once a regime is reached where one parameter, such as precipitation rate, dominates and this can obscure effects of other more subtle parameters. Secondly, a type of measurement equifinality is observed if a structure is summarised by a particular measurement; it is possible for simulated microbialites with very different internal structures to yield similar values for some measurements, or for measurements to be insensitive to parameter changes until a phase transition occurs in the output. Strong absolute equifinality, where distinct parameter sets give exactly identical outputs, has thus far not been found in this model because of the complexity of the parameter space, the interactions within it, and the stochasticity of the model. One might anticipate that by halving one parameter and doubling another, or some conceptually equivalent action, it would be possible to create a structure which is absolutely identical to the original, but this is not observed despite some effort to search for it.

Figure 6 details how these observations play out. Figure 6a shows how increasing precipitation amplitude leads to a regime where surface roughness is suppressed and structures merge together, with Figure 6b,c showing how a large change in attraction distance makes only a small contribution to the final outcome in this regime.

**FIGURE 6:**
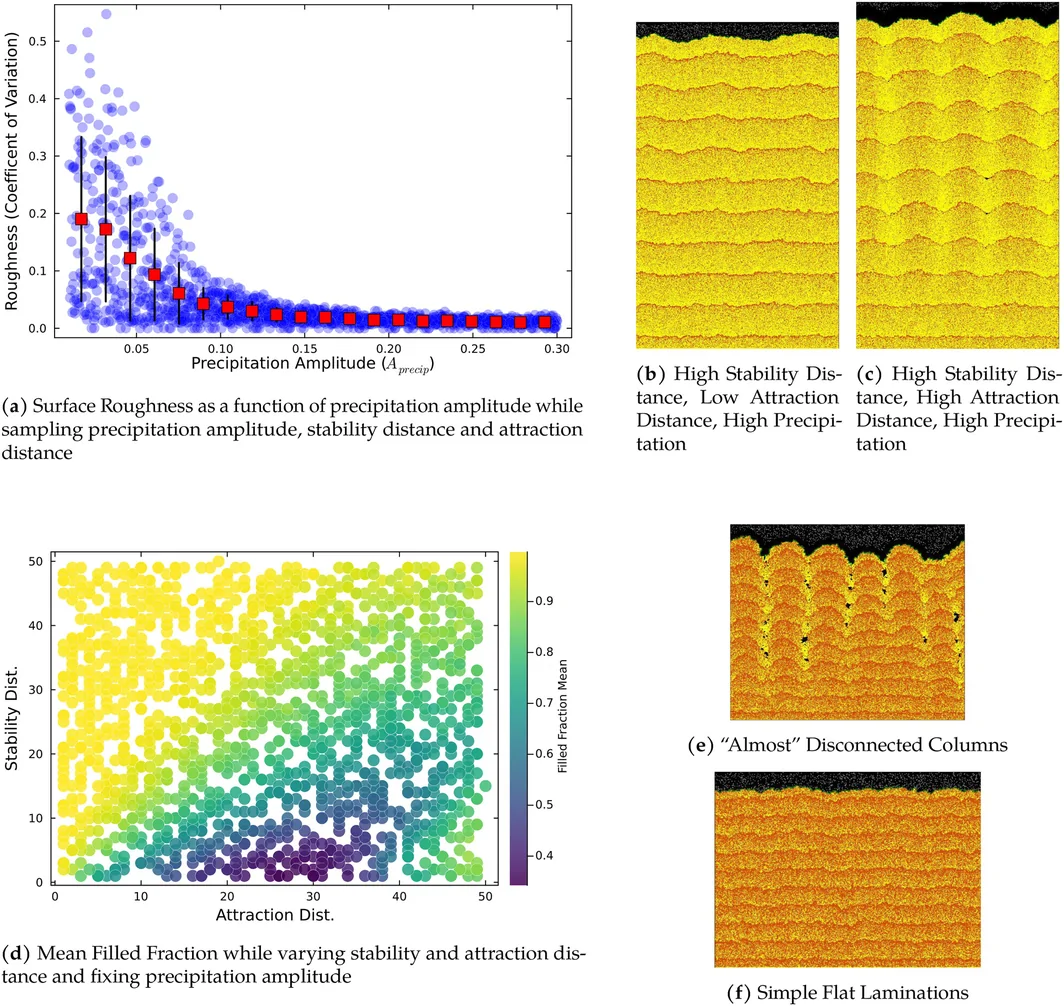
Quantitative measurements and equifinality. (a) Suppression of surface roughness and detail by increasing precipitation. (b, c) Demonstratation of how distinct columns that may have been created by varying attraction distance are suppresed by high precipitation. (d) Variations in mean filled fraction across parameter space. (e, f) Nuances of structure that can be missed by this measurement.

We can also demonstrate measurement equifinality in Figure 6d–f where we see two examples of two structures that have almost identical mean fractions of each row filled. This parameter (which can successfully detect the emergence of columns and branching) is insensitive to the difference between flat laminations and the ‘almost’ splitting columns. This could be avoided by choosing different measurements, but the key point is that reducing the structure to a set of measurements inevitably loses information. Hence, relying only on quantitative measurements can miss important nuances.

This result has implications for biogenicity assessment using quantitative measurements. Care must be taken to ensure that the chosen measurements adequately capture the nuances of the structure. Moreover, biogenicity determination using methods that depend heavily on a single parameter is vulnerable if other factors can hide the effects of that parameter.

## 5 Discussion

### 5.1 Extensibility

One key strength of this work is accessibility. The code for this model is written in a high‐level, open‐source framework that ensures both ease of use and computational efficiency, lowering the barrier to entry for further development and experimentation. Additionally, we offer the first interactive simulation tool, which is intended to develop users' intuitions about structure formation, inspire new hypotheses about microbialite growth, and serve as a potential outreach or teaching aid. This model is presented at an opportune time. We expect that agentic AI will excel at building variants of this model from the starting point and high‐level code framework offered in this study.

A closely related strength is extensibility. As this is a high‐level minimal model, there are vastly many possible extensions to this work. We include the heuristic conical‐like stromatolite morphospace in the Supplementary Information to demonstrate one possible extension while also highlighting the additional complexity and parameter creep that may occur. To reduce complexity in this study, we deliberately omit additional factors, such as a rising tide of background sedimentation, which could interact with the structure, as this would complicate our morphological measurements and increase parameters further. However, incorporating such processes may be necessary to simulate certain microbialite formation scenarios more accurately, particularly those where ‘normal’ background sedimentation competes with the growth of microbialites.

Another natural extension of the current model would be a three‐dimensional implementation. True microbialites are of course three‐dimensional objects and include structures that depend on this three‐dimensionality, as illustrated by Curtis et al. (2021) in one particular context. This would require a relatively minor update to the simulation logic, although changing the dimensionality may affect the type of structures formed. Extensions to a third dimension could be accompanied by implementation of more realistic hydrodynamics, to show the roles that currents and tides may play in the formation of microbialites. This would require solving for the fluid velocity field given the morphology and boundary conditions and advecting the sediment according to this. This could also dovetail with implementation of erosion and scouring dynamics to modify the structure over time in response to fluid flow, an important factor in microbialite preservation and morphology.

Other disruptions of the structure could also be added, such as gas bubbling, physical disturbance of the mat by other organisms, or slumping due to mechanical instability. A range of modifications could be made to move beyond the simple model of the sediment dynamics to incorporate more realistic sediment behaviour and intra‐sediment interactions (e.g., capturing sediment angle of repose). Some attempts were made during the early development of our model to have the sediment behave in ‘sticky’ ways only when precipitating agents were nearby, simulating the need for EPS to bind the sediment. However, this was removed for simplicity. A more bespoke implementation which aims to link geometry of sediment structures to biogenicity would also be of value (Tice et al. 2011; Frantz et al. 2015) although outside the scope of the present study.

Furthermore, the growth dynamics could be replaced with a more realistic representation of the drivers of precipitation, for example by simulating the microbial metabolic pathways (e.g., photosynthesis, sulfate reduction) that drive changes in local geochemistry. Finally, real microbialites are often significantly altered during diagenesis, with potential overprinting of their primary structure. A future extension of the model could incorporate a simplified diagenetic filter to approximate post‐depositional changes such as recrystallisation, compaction or cementation, further bridging the gap between simulation and observed field structures.

Another modification which could be made is to introduce a structured temporal variation to the parameters beyond what can be done with the interactive simulation. This could reflect a variety of variations in the local environment (such as tidal cycles, seasonal changes, hydrothermal pulses or changing microbial community composition). Such temporal variation is a suspected driver of the structure of real microbialites (Reid et al. 2024; Petryshyn et al. 2012; Szulc and Smyk 1994). We could also easily incorporate additional classes of sediment or precipitate, which may have different dynamics and interactions, potentially encompassing a larger variety of scenarios in which laminations and other structures form. These could represent, for example, varying grain sizes, mineralogies (e.g., carbonates vs. sulfates) or biologically vs. abiotically mediated phases.

The ultimate goal of any modelling procedure may be to link the model parameters to the real‐world parameters. However, we do not wish to overstate what can be done in this regard. For any natural structure, simulations with plausible parameter values may show that both biotic and abiotic processes can reproduce its salient features but that contextual knowledge of the environment in which the structure is found strongly favours one interpretation over the other. A limitation of our generic aggregative approach is that it would need to be made much more specific in order to capture differences in behaviour between biotic and abiotic systems, and at the same time more complex and less generalisable. It may be that one could compare real and generated structures and use the similarities to attempt to tune model parameters to match physical reality, but this would be a non‐trivial task outwith the scope of this work. One problem is that a given layer thickness is both a function of growth rate and the time it was allowed to grow, with both needing to be constrained simultaneously. There is also the problem that the simulation units used in the model cannot be trivially converted into real‐world physical units. This is a significant challenge, as the simulation parameters are an aggregate of many factors, and DLA leads to self‐similar behaviour. There are ample possibilities for future work in this regard. It would also be worthwhile to relate the quantitative metrics used to describe the structures in this work to those commonly applied in field‐based and image‐based studies of microbialites.

### 5.2 Implications for Biogenicity and Inference of Generative Process

It is well understood that morphology alone is an ambiguous indicator of biogenicity (García Ruiz et al. 2002; McMahon and Cosmidis 2022). Our model demonstrates how relatively simple mechanisms, when properly arranged, can lead to a wide diversity of microbialite morphologies. This supports the claim that morphology is ambiguous in the following way: in our model, the parameters are a highly simplified abstraction of the underlying processes. From the perspective of the model, it does not matter what causes a particular parameter value or even what substance the ‘sediment’ and ‘precipitate’ represent. This implies that a given morphology could be the result of a range of biological or abiotic processes or some combination of the two. This is a form of ‘external equifinality’ where the morphology is the result of simple interacting processes based on few parameters, which may represent a wide range of physical mechanisms.

Hence, a principled extension of this work would be to explore how models of this type (or extensions) could also simulate abiotic structures that are morphologically similar to microbialites such as the ‘paint stromatolites’ of McLoughlin et al. (2008) or the formation of wind‐driven digitate sinter structures (Gong et al. 2022) similar to objects observed on Mars (Ruff and Farmer 2016).

Overall, this study suggests that multiple, distinct physical or biological processes may be effectively captured by the same simple model mechanism, reinforcing the point that morphology, while informative, is not sufficient on its own to resolve biogenicity without further contextual (e.g., geochemical) evidence.

### 5.3 Geobiological Implications

This work has focused on a subset of the most influential and directly interpretable parameters, selected for their relevance to previous literature (Dupraz et al. 2006). Of course, there is a whole range of behaviour that may be extracted from varying other parameters; however, we limited our scope to a concise set for concision, clarity and interpretability. The model presented here does not provide a detailed account of every aspect of microbialite formation but complements the existing literature by emphasising the need for models to consider internal structure. The results are promising enough to motivate further exploration of agent‐based approaches in this field. It is crucial that models try to move beyond gross morphology and begin addressing internal layering and composition.

Here, we have focused on external morphology and broad categories of internal fabric. In line with the stromatolite literature (Reid et al. 2024; Petryshyn et al. 2012; Szulc and Smyk 1994), we have assumed that layering in real stromatolites might result from a fixed external periodicity, such as seasonal or climatic cycles, and successfully reproduced this in the model. However, the origin of laminations and the nature of periodic structures observed in stromatolites are fundamental questions deserving more sustained attention. Hence, we plan to dedicate a future follow‐up paper solely to addressing these questions.

Our model successfully reproduces domal and weakly branched columnar architectures, which are characterised by high syn‐depositional inheritance and uniform, isopachous laminae (Figures 2d and 3d). In sedimentary systems, isopachous geometry may reflect continuous or episodic in situ precipitation across the entire growth front, where mineral accretion rate is uniformly distributed along the surface (Reid et al. 2000; Riding 2008; Lepot et al. 2008; Sforna et al. 2014). This contrasts with detrital‐dominated growth, where grain accretion is strongly influenced by gravity and local slope angle, leading to dynamic instability and widespread branching (Dupraz et al. 2006). Our model implies that smooth, isopachous stromatolitic fabrics may emerge when periodic precipitation is coupled with sedimentation. This process does not require complex hydrodynamics or pre‐existing substrates. Furthermore, this initial isopachous precipitation may stabilise the structure, making it mechanically rigid and enhancing the taphonomic preservation of the microbialite, resisting current scouring before burial.

This model may provide geobiological insight regarding the progressive accumulation of precipitate within intercolumnar spaces during periods of reduced detrital flux or near‐constant precipitation (Figure 5), something which cannot be visualised in previous models (Dupraz et al. 2006). In modern and ancient microbialites, the early syn‐depositional lithification of intercolumnar voids is potentially important for the preservation of complex, multi‐branching architectures. Uncemented columnar structures like those simulated by Dupraz et al. (2006), or those interspersed solely with trapped sediment grains, may be mechanically unstable and vulnerable to wave scouring, hydrodynamic erosion or syn‐depositional collapse. By contrast, in situ precipitation that fills intercolumnar porosity, as seen in our model, may provide structural reinforcement, locking fragile branches into a rigid carbonate framework before deeper burial. This stabilisation process may increase the potential for preservation of delicate microbialite morphologies.

## 6 Conclusion

We have presented an agent‐based model of microbialite formation, which produces for the first time a wide range of morphologies, both internal and external, while being accessible interactively. This model demonstrates that diverse morphologies can originate from simple rules coupled to external periodicity, highlighting the ambiguity and non‐uniqueness of morphology, as many biotic or abiotic processes may map to these rules. We also highlight how our setup naturally facilitates quantitative analysis of the model outputs, and offer several suggestions for extensions of the model, one of which we demonstrate via a conical heuristic in the Supplementary Information. Although deliberately abstracted and high‐level, our model captures essential aspects of microbialite morphogenesis and highlights the value of agent‐based approaches to explore structure–process relationships. We hope that this model provides a useful resource and impetus for renewed efforts in the modelling of microbialite formation, ultimately leading to a better understanding of these important objects, of the roles of biotic and abiotic processes in their formation, and of signatures by which microbialites might be distinguished from fully abiotic structures.

## Funding

The authors acknowledge the support of the Human Frontier Science Program under grant number RGP006/2024.

## Conflicts of Interest

The authors declare no conflicts of interest.

## Supporting information


**Figure S1:** Modified simulation in an attempt to make conical‐like stromatolites. Increasing the conical scale parameter leads to a stronger preference for vertical growth while spreading and internal morphology are modified by the precipitation amplitude. Precipitation in yellow, sediment in red.
**Figure S2:** Subset of quantitative structural trends caused by parameter variation in parameter sweep one. Each point is a unique simulation. Red squares are the mean values of the local binned region, with error bars one standard deviation within these bins.
**Figure S3:** Subset of quantitative structural trends caused by parameter variation in parameter sweep two. Each point is a unique simulation. Red squares are the mean values of the local binned region, with error bars one standard deviation within these bins.
**Figure S4:** Correlation matrices obtained when fixing and sampling different regions of parameter space. Varying precipitation growth rate suppressing trends visible in the stability and attraction distance only correlation matrix. Correlation values represent the Pearson correlation coefficient.
**Table S1:** Default parameters used in the simulation.
**Table S2:** Non‐default parameters used to reproduce Goose Egg Formation flat laminations.
**Table S3:** Non‐default parameters used to reproduce Nash Fork Formation Stromatolite.
**Table S4:** Non‐default parameters used to reproduce linked‐columnar stromatolite.
**Table S5:** Non‐default parameters used to make branched domal microbialite from the Howe Limestone Member formation.
**Table S6:** Non‐default parameters used in the non‐conical morphospace.
**Table S7:** Non‐default fixed parameters used in the conical morphospace.
**Table S8:** Non‐default parameters used in the quantitative parameter sweeps.
**Table S9:** Non‐default video parameters (does not include stability and attraction distance values, see text).

## Data Availability

All data, including images of all quantitative sweep microbialites, example videos of dynamics, the code and instructions for how to run the interactive model can be found at https://github.com/nrodgers1/Microbialite_simulator. This was permanently archived at https://doi.org/10.5281/zenodo.23392551.
