# Supplementary material for: Agent‐Based Modelling of Microbialite Formation Through Sedimentation and Precipitation Dynamics
Source: Geobiology. 2026 Nov 4;24(6):e70071. doi: 10.1111/gbi.70071 (PMC13736403; doi:10.1111/gbi.70071)
Supplement: Supplementary file 1 [file GBI-24-e70071-s001.pdf]

Supplementary Information - Agent-based Modelling of Microbialite Formation through Sedimentation and Precipitation Dynamics

Supplementary Information

In this supplementary information, we give detailed descriptions of all parameters, list the parameters used, and describe the quantitative measurements made. We also give more details on the running of the interactive model, the model implementation, and model extensibility.

Contents

A Tutorial on the Interactive Simulation **3**
- A.1 Video of Simulation Runs 3

B Parameter Descriptions **3**
- B.1 Agent Properties 3
- B.2 External Forcing 4
- B.3 Model Setup Parameters: 5

C Conical Structure and Model Extensibility **5**

D Quantitative Analysis of Structure **6**
- D.1 Measurements Made 6
- D.2 Parameter Values Tested 7
  - D.2.1 Sweep 1 8
  - D.2.2 Sweep 2 8
- D.3 Quantitative Analysis - Parameter Sweep 1 8
- D.4 Quantitative Analysis - Parameter Sweep 2 9
- D.5 Correlation between Input Parameters and Measurements 11

E Additional Model Details **11**
- E.1 Initial Conditions Setup 11
- E.2 Morphospace Setup 11

F Parameter Values Set **13**
F.1 Parameters Used for Comparisons to Real Objects 14
F.2 Morphospace Parameters 14
F.3 Quantitative Sweep Parameters 14
F.4 Video Parameters 17

G Quantities Measured **18**

A Tutorial on the Interactive Simulation

Interactive simulations require installation of the `Julia` programming language and the relevant packages; however, when this is installed, it should be possible to run with minimal interface with the actual code. `Julia` language can be installed with little effort by following the detailed documentation of the language provider `https://julialang.org/`.

We provide two ways to launch the interactive interface: (1) by using a Jupyter Notebook and (2) by running the executable `.jl` file directly. In both cases, the relevant packages, `[Agents, LinearAlgebra, Random, StatsBase, GLMakie]`, must be installed. The `.jl` file is configured so that the relevant packages are automatically checked for and installed. This means that the interactive simulation can simply be launched by typing `julia -i "path to .jl file"` into your system terminal or command prompt and the interactive simulation will begin. The sleep function should be set to zero if you do not want pauses between time steps, and the number of steps per frame can be set by changing the `dt` parameter. The simulation will begin with default settings, where the initial conditions and range of variables on the sliders are can be changed by updating the code. The interactive code file is minimal, so all relevant variables can be found at the end of the file. Additional details can be found in the code repository.

A.1 Video of Simulation Runs

Similarly to the interactive interface, there is a way to directly generate videos from the `Agents.jl` simulations. In the code repository, we attach a few videos visualising runs and attach a notebook which can be used to generate such videos. We select 15 time steps per frame to decrease the file size. Full parameter lists are given in the parameters section.

B Parameter Descriptions

In this section, we describe each of the parameters used in this work in more detail. The model contains the following parameters, broken into three categories. There are 21 parameters in total, some of which are physically important while others are only of computational necessity as detailed below. In principle, to reduce parameter count and enhance interpretability in some agent-based modelling contexts, one could attempt to non-dimensionalise or couple parameters. For example, one could set the stability distance to scale with attraction distance, or define sediment amplitude relative to grid width. In this study, we choose not to do this for several reasons. We do not operate in a regime where a single pixel discretisation is small compared to the quantities of interest. The raw values of parameters such as search radii matter for the computational runtime, and influence the speed observed when running the interactive simulations. The independent values of some parameters, like the stability and attraction distances, give independent control of the structure, not fully captured by their ratio.

B.1 Agent Properties

- Precipitation Amplitude - Maximum probability for a precipitating agent to add precipitate to a neighbouring cell and spread. This value multiplies any periodic function. This controls much of the final morphology and the appearance of layers. This parameter can be thought of

as aggregating the physics, chemistry, and biology that control the rate of precipitation, such as pH, available carbonate, temperature, and microbial activity.

- Removal Probability - Probability for the precipitating agent to be removed at each step. This could be interpreted as representing a local disruption of the microbial mat or fluctuations that prevent precipitation at a location.
- Precipitation Weights - Vector of length 3 which sets the relative preferences for upward, horizontal, or downward precipitation. This relates to the hypothesis that microbial activity may tend to push growth upwards for photosynthesis. This hypothesis may not be relevant in all scenarios, so this parameter can be modified.
- Stability Distance - Stability distance for sediment agents. This is a parameter derived from the DLA that represents the ability of the sediment to settle and fall to a stable position. This would depend on the environmental conditions and stickiness of the surface (e.g., the adhesion of the mat driven by EPS).
- Attraction Distance - Attraction distance for sedimenting agents. This is a parameter inspired by DLA that drives the morphology. This represents the distance at which the sediment agents stick to the structure. This is related to the adhesive properties of the surface (e.g., microbial mat) and the environmental conditions.
- Recolonisation Probability - Probability of introducing a precipitate agent where the sediment lands. This can be thought of as the probability that microbial mat growth covers newly arrived sediment or the sediment acts as a nucleation site for potential abiotic precipitation.
- Down speed - Fixed size of vertical random walk steps. This down speed represents the step size of the biased random walks of the particles downwards. Large down speeds approximate ballistic motion, which can prevent the formation of some morphologies.
- Side speed - Fixed size of horizontal random walk steps. This parameter controls how fast the particles move side to side when doing the random walk. We could also bias this if desired to represent a flow.
- Conical Scaling - The parameter that governs how much the growth rate is rescaled depending on the number of solid neighbours above the agent. This can be set to zero to disable this behaviour, with positive values leading to more tip dominated growth.
- Prevent Overhangs - This boolean value sets the kind of overhanging allowed by the model. This can prevent mushroom shapes with a flat overhang, or allow them.

B.2 External Forcing

- Sediment Amplitude - The maximum number of sediment agents added at each step. This causes the structure to grow by sedimentation and must be a whole number. A rescaled version of this could also be constructed, which varies depending on the simulation grid width to maintain the sediment density.
- Period Control Parameter - Scaling of oscillations for given periodic functions that affect the sediment and precipitation. This parameter is defined mathematically in the main text. It can be interpreted to represent a variety of environmental periodic behaviours or timescales.

- Precipitate Agent Period Scaling - Multiplicative constant that sets how much the precipitate cycles scale to the sediment cycles. See the main text for mathematical details.
- Precipitate Agent Period Offset - Offsets the cycles of the precipitate agents by a given constant. See the main text for mathematical details.
- Sediment Forcing Function - This is the choice of function to set the oscillations. This must be positive, so we give the following options: $\max(\cos x, 0)$, $\cos^2 x$, or $|\cos x|$.
- Precipitation Forcing Function - This is the choice of function to set the oscillations. This must be positive, so we give the following options: $\max(\cos x, 0)$, $\cos^2 x$, or $|\cos x|$.

B.3 Model Setup Parameters:

- Initial Conditions - Choice of whether to set a flat layer, patches, triangular, a mound, or sinusoidal initial substrate.
- Model Width - Width of the simulation grid. The grid is horizontally periodic. A key detail is that the sediment amplitude and grid width will interplay as this affects the density of sediment per cell.
- Model Height - Height of the simulation grid. This effectively sets how long the simulation can run before the solid fills the grid, and the number of layers visible.
- Distance Metric - Can be set in Agents.jl. We choose to use Euclidean distances for this work, but users could also choose Manhattan if preferred.
- Initial Precipitate Agent Density - The density of initial precipitate agents placed on the available substrate.

C Conical Structure and Model Extensibility

Some fossil and living stromatolites are characterised by a conical external morphology. They have been well modelled, as in the work of [1], and are similar in gross morphology to abiotic objects such as stalagmites [2]. In this work, we do not reproduce the specific physics of models such as those applied in Petroff et al. [1] and Szymczak et al. [2]. However, we show how our model, which does not natively produce conical morphologies can be relatively easily modified to create heuristic conical-like structures. This provides a minimal model of cone formation coupled to our model of internal microbialite structure.

Conical structures, be they biotic or abiotic without overhangs, are generally formed when the apex of the structure grows faster than the surrounding areas, producing a leading point which grows ahead of the rest of the structure. This may be considered a result of diffusion-driven processes [1] or shadowing, depending on which mechanisms are applicable to the object in question.

The heuristic we add to the model is as follows: When assessing the likelihood for precipitation, we rescale it by an exposure factor. We do this by multiplying the precipitation probability by this factor, which is defined as $(1 + n_{\text{above}})^{-c}$ where c is the conical scale parameter, which must be non-negative and n_{above} is the number of neighbours above. This is largest when the agent has no neighbours in the row of cells above, at a tip, but reduced when the agent has any neighbour blocking

it. When c is zero, we see no effect and higher values of c make the cone more peaked. Additionally, we modify the preferred growth direction to increase the preference for upward growth. These changes allow various coniform structures to occur, with the precise morphology depending on the interplay of all parameters. To maintain physical plausibility and prevent unrealistic overhangs (e.g., mushroom-shaped structures), we also enable a flag which can be turned on to prevent horizontal overhangs by allowing growth only if there is a supporting structure directly or diagonally below.

Conical structure growth depends on the initial conditions, which shape how the central tip emerges. In order to create centred conical structures, we start the cones growing from small triangles to allow the tip to grow at the intended location. This also prevents impassable walls from forming at the edge of the initial conditions due to overhangs at $t = 0$. The shape of the cone is sensitive to the curvature of the initial structure, as the growing layers are influenced by the previous layer, with this influence extending back to the initial shape to various degrees, depending on the parameter combination.

To maintain the growth of conical structures, we require a very low (or zero) precipitating agent removal probability and a recolonisation probability close to one, because removal of the one cell at the growing tip can suppress the emergence of the conical structure. Our cones may display fractal-like behaviour, where a large cone forms with a surface made of smaller conical features.

In our model, conical stromatolites occur in the regime dominated by mineral precipitation, where the conical form is the result of the preferred direction of precipitation and not directly caused by the actions of sediment. Depending on the interplay between the parameters, layers may or may not appear in the conical structures. One challenge in making even small additions to a model like this is the potential cascade of effects from the changes. In this case, modifying the precipitation probability by the exposure factor reduces this key control parameter, which then changes the range in which other changes in morphology occur. Hence, we do not dwell on the specifics of the conical-like structures, but show how our model can be modified to capture various effects and parsimoniously reproduce key structures.

An example of a heuristic-conical morphospace is given in figure S1, (detailed parameters given in table S7). The extent to which these structures appear conical depends on both the conical scale parameter and the precipitation amplitude as shown. However, as the model lacks settling dynamics where the structure relaxes, we do not make true triangular cones but end up with cones that are more vertical and pine-cone-shaped. This demonstrates how the model can be extended in some cases quite easily, while some other cases would require a more fundamental rewriting of the logic.

D Quantitative Analysis of Structure

Previous related work on the modelling of stromatolites [3] has been mostly qualitative. However, we propose linking the input parameters with variables that can describe the output structures. This allows a more precise indication of where in parameter space changes in morphology can occur and which parameters control various aspects of the structure.

D.1 Measurements Made

We aim to measure a reasonable range of properties which are computationally efficient and summarise the morphology of the objects. Thus we aim to select quantities which minimise between-measurement correlation aside from the case of obvious parameters like the mean and maximum height, for example. The selected set of measurements assumes that we are starting from flat initial

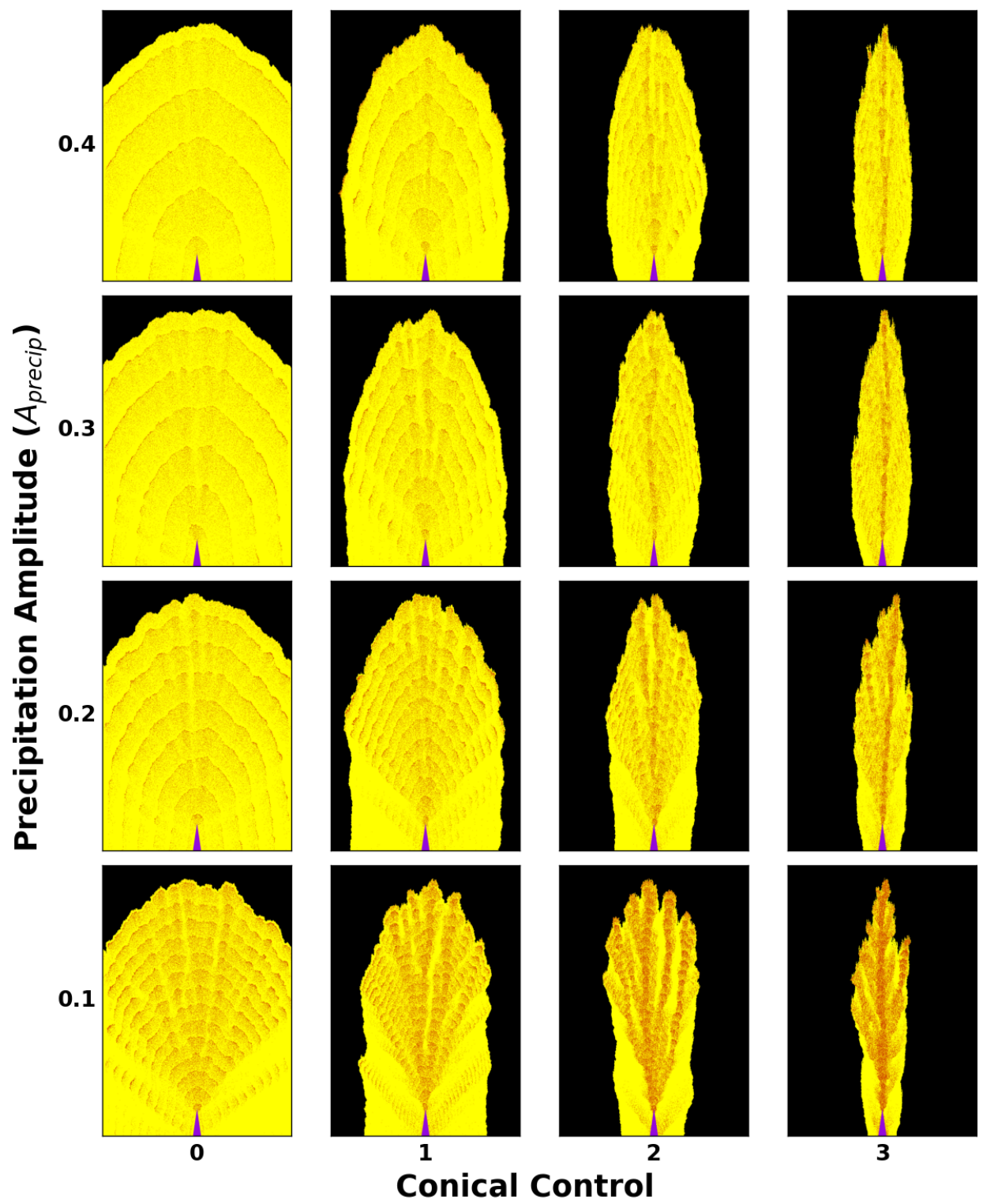

Figure S1. Modified simulation in an attempt to make conical-like stromatolites. Increasing the conical scale parameter leads to a stronger preference for vertical growth while spreading and internal morphology are modified by the precipitation amplitude. Precipitation in yellow, sediment in red.

conditions and that we have uniform horizontal behaviour throughout the model for simplicity. If one implemented localised initial conditions or other parameters that break the horizontal uniformity of the structures, then modified measurements would need to be used to account for this. A full list and description of the eleven measured quantities is given in section G.

D.2 Parameter Values Tested

When quantitatively studying morphology we use two parameter sweeps focusing on precipitation amplitude, stability distance, and attraction distance. We do one sweep with varying precipitation amplitude and one with this parameter fixed. This allows us to see in more detail the impact of structural parameters; the precipitation rate can obscure other trends as it has a strong control over morphology and can overwhelm the other parameters. All other parameters are fixed for this parameter sweep to the values given in appendix, F.3.

In order to sample the parameter space and to have control over the number of samples while fixing the parameter range, we use Latin Hypercube sampling to generate 1500 samples, with 10000 time steps per sample. Latin hypercube sampling generates random samples such that the space is partitioned by the samples and samples are approximately evenly distributed across the space. Each

simulation is an independent process, so we can run this in parallel using `Julia` multi-threading and generate large numbers of samples efficiently on a laptop. We generate the same number of samples for each sweep using Latin Hypercube sampling irrespective of the dimensionality of the sampling space, with the sampling plan floored to integers for parameters which require this.

Due to the large number of measurements made, there are a large number of combinations of measurements and parameters that could be plotted. In the text, we give a small selection of these and in the supplementary material attach all the pairwise combinations of parameter plots, the raw data and `.jpg` image for each simulation, and a `.csv` of all values across simulations.

D.2.1 Sweep 1

In the first sweep, we sweep the stability and attraction distance in the set $[1, 50]$ and vary the precipitation amplitude parameter between [0.01,0.3]. Increasing the precipitation amplitudes to larger values outwith this range tends to fill most of the grid with mainly precipitate.

D.2.2 Sweep 2

In the second sweep, we wish to isolate the effects of stability and attraction distances, so we set the range for each as integers in the set $[1, 50]$ and fix the precipitation parameters.

D.3 Quantitative Analysis - Parameter Sweep 1

The results of the first parameter sweep are given in figure S2, which shows how precipitation rate controls various aspects of the morphologies. Figure S2a, shows the relationship between the fraction of the solid object made up of precipitate and the input precipitation amplitude. As expected, there is a strong correlation, with an almost linear trend, between the input precipitation amplitude and the observed precipitate, showing that the model behaves as intuitively expected.

The effect of precipitation amplitude on the structure can be quantified by using the gzip compression ratio, which compares the file-size of the uncompressed object to the file-size of the compressed object using the gzip algorithm. gzip compressibility is a very simple way (although limited) to assess the complexity. It gives a sense of how easy the object is to compress, the extent of internal uniformity or repetition, and how far it is from random noise (more difficult to compress). Figure S2b shows that as we increase the precipitation amplitude, we decrease the compressibility because we create more mixing of precipitate and sediment, making it harder for the algorithm to compress. In the low-precipitation regime, there is still a large variation in compressibility, as the different morphologies all compress to varying degrees due to their observed complexity. Compression-based quantification of microbialite complexity has been used previously in the work of [4, 5].

The dependence of the maximum height of the generated object on the stability distance and the precipitation amplitude is given in figure S2c. The maximum height tends to increase as one increases precipitation amplitude, as more solid structure is formed. However, the tallest structures occur when the stability distance is low and the sediment particles cannot rearrange, sticking directly to the tallest branches and so driving the structure upward.

The interplay between precipitation amplitude and attraction distance on roughness (measured as the coefficient of variation of the object height per column) is shown by figure S2d. This shows how high attraction distances promote branching, leading to higher measured roughness but that

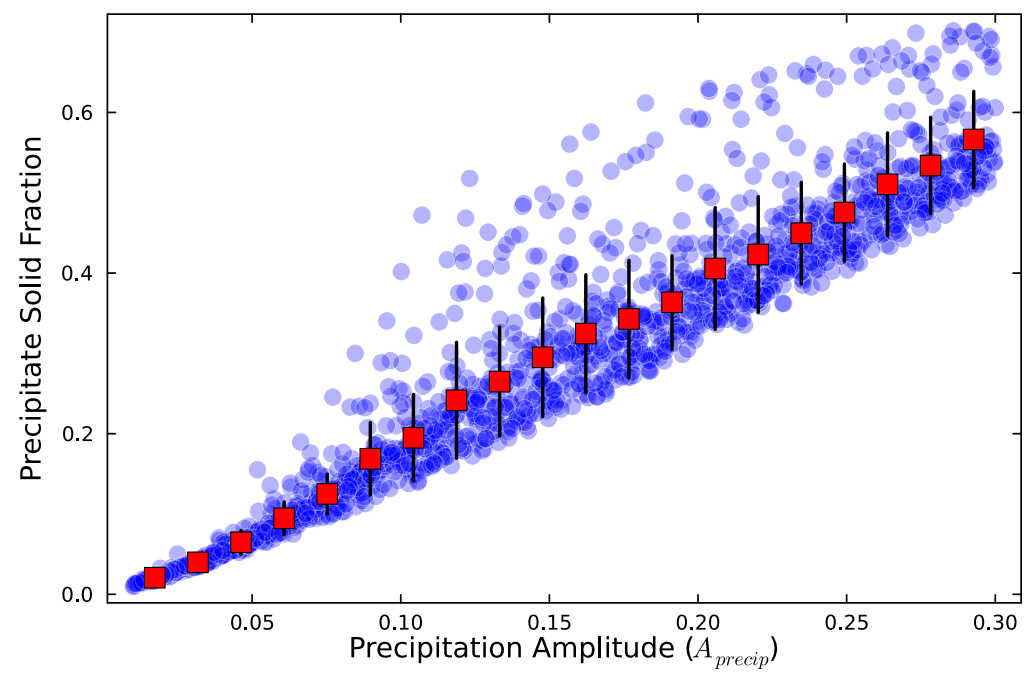

(**a**) Increasing precipitation amplitude leads to a higher fraction of precipitate as expected

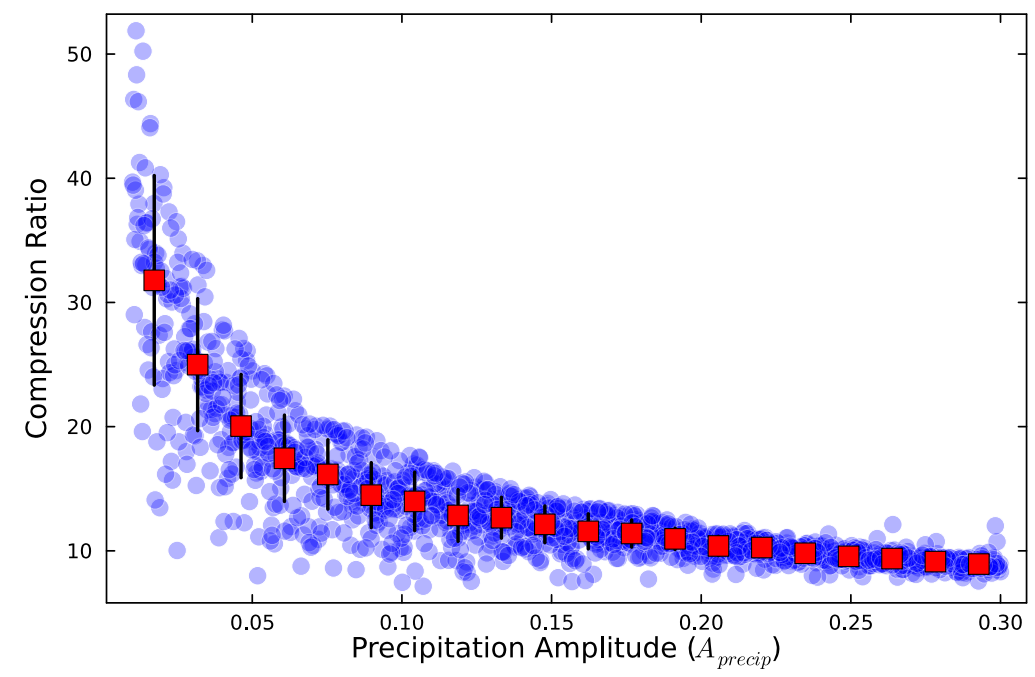

(**b**) Variations in measured structural complexity with precipitation amplitude

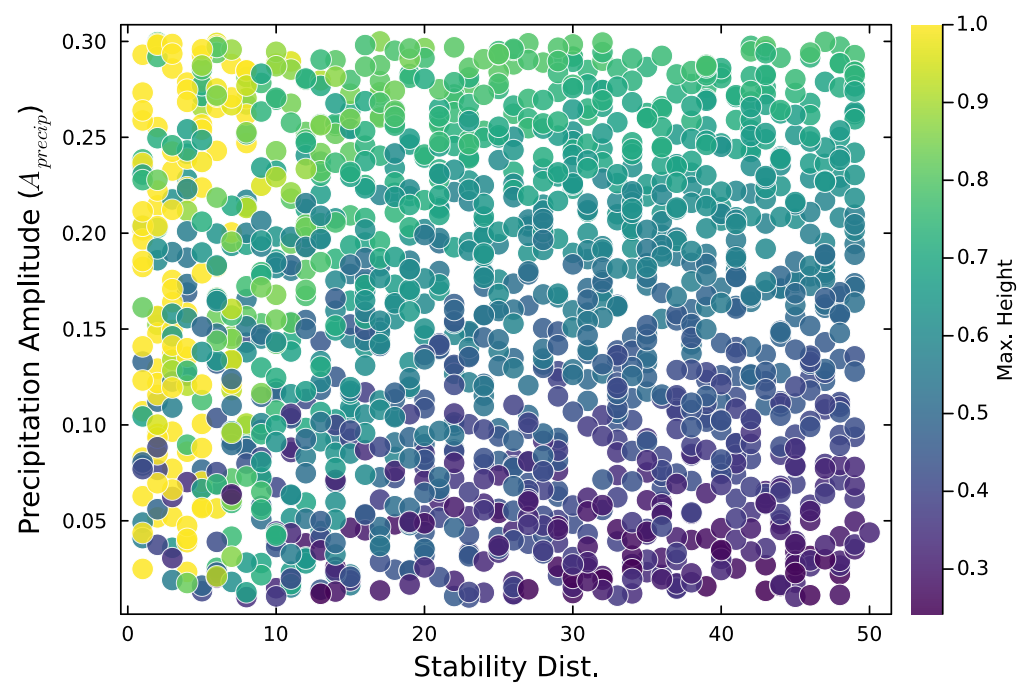

(**c**) Interplay between stability distance and precipitation amplitude on maximum height

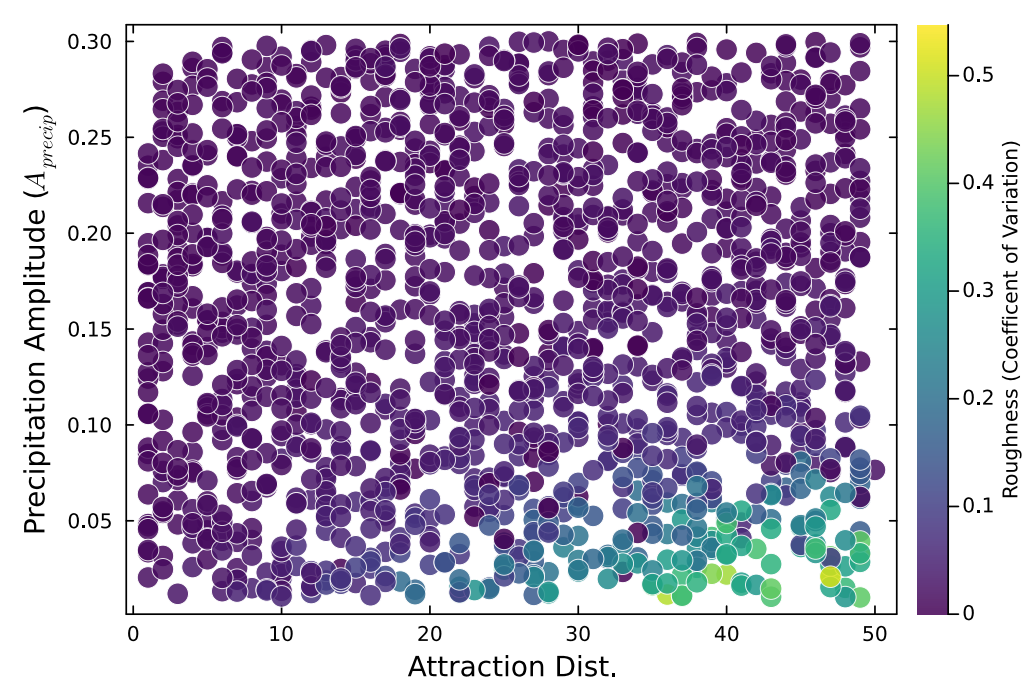

(**d**) Variation in roughness (measured by coefficient of variation) with precipitation amplitude and attraction distance

Figure S2. Subset of quantitative structural trends caused by parameter variation in parameter sweep one. Each point is a unique simulation. Red squares are the mean values of the local binned region, with error bars one standard deviation within these bins.

this can be suppressed by high precipitation rates, which fill in the gaps between branches. This reveals how certain aspects of the morphology can be suppressed by high precipitation regimes.

D.4 Quantitative Analysis - Parameter Sweep 2

In this parameter sweep, figure S3, we more clearly expose the interactions between stability and attraction distance in the formation of the morphology by fixing the precipitation amplitude.

Figure S3a shows how roughness (measured by the coefficient of variation of the surface) correlates with the attraction distance due to the tendency for larger attraction distances to lead to branching, where the stability distance allows. This matches the trends observed when viewing the qualitative morphospaces of stability and attraction distance.

Similar trends can be captured by figure S3b, which shows how the mean filled fraction in each row of the object varies with stability distance. The mean filled fraction approaches one when all the rows are filled and we have a uniform solid structure. It is smaller when we have a columnar

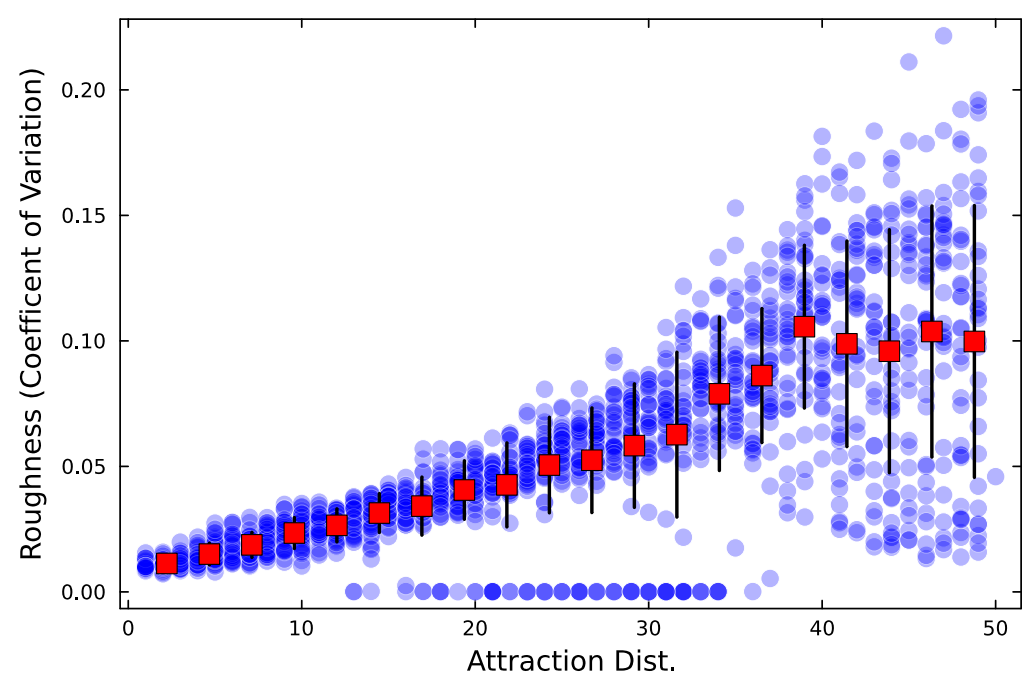

(**a**) Increasing in branching caused by an increase in attraction distance results in an increase in roughness (measured by coefficient of variation)

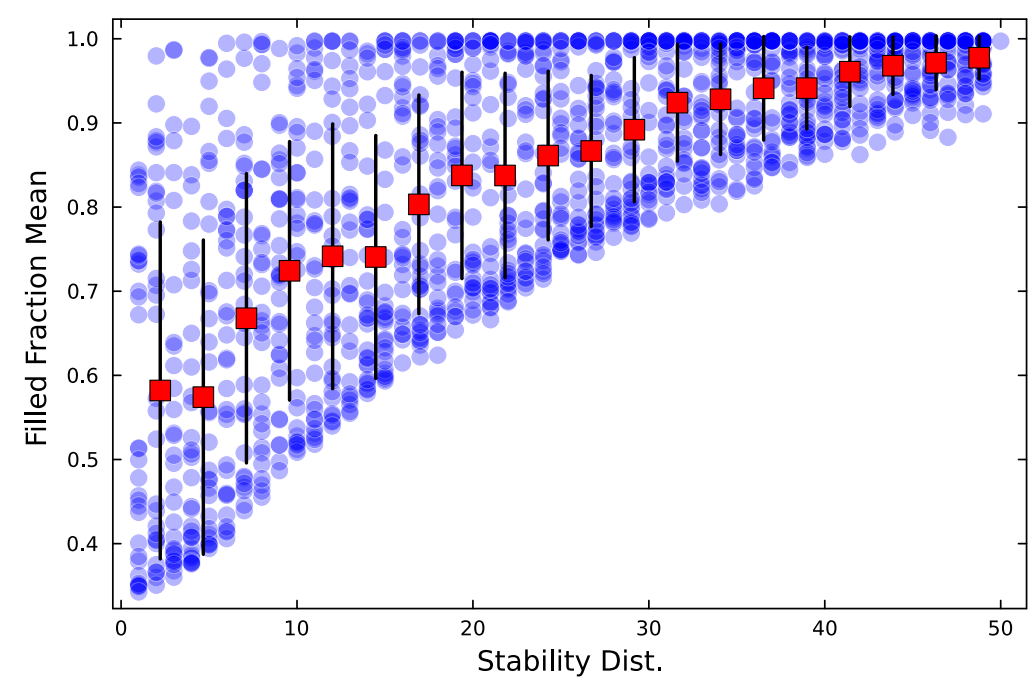

(**b**) Increasing stability distance leading to an increase in mean filled fraction (horizontally across rows)

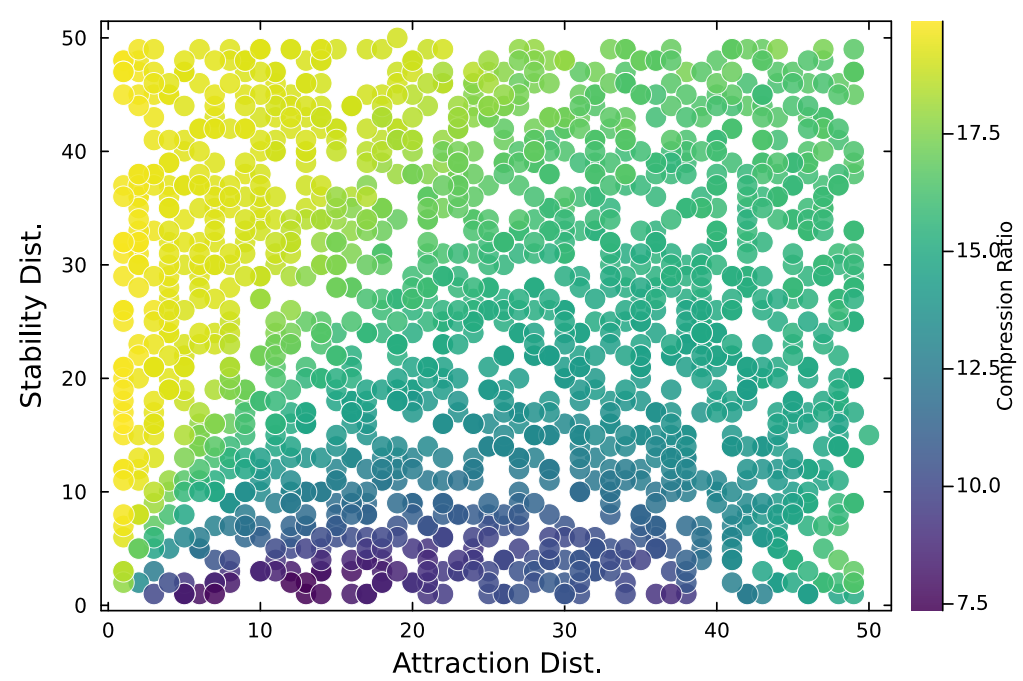

(**c**) Interplay of stability and attraction distances on complexity as measured by the compressibility

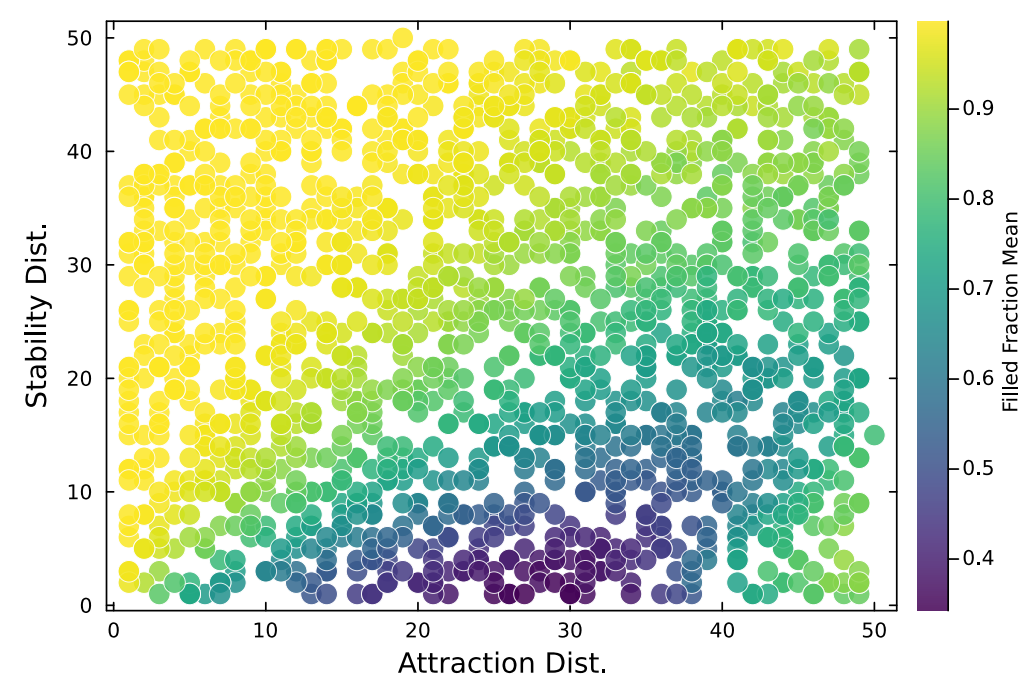

(**d**) Mean filled fraction with stability distance and attraction distance showing distinct types of morphologies created.

Figure S3. Subset of quantitative structural trends caused by parameter variation in parameter sweep two. Each point is a unique simulation. Red squares are the mean values of the local binned region, with error bars one standard deviation within these bins.

or branching structure with gaps. For larger stability distances, the number of columns decreases until the sediment settles across the domain uniformly, whereas smaller stability distances lead to a variety of filled fractions depending on the density of the branching caused by the attraction distance.

In agreement with the qualitative impression given by the morphospaces, the variation in compressibility highlights the array of morphologies that result from varying the stability and attraction distance, with different regions of the plot showing distinct behaviour (figure S3c). For low attraction distance and intermediate-to-high stability distance, we see higher compressibility due to the regular planar structures formed. For higher attraction distances with low stability distances, we see reduced compressibility from the emergence of branching structures. Columnar structures formed when both distances are larger yield intermediate compressibility. Eventually, very large attraction distances also lead to column-like structures as sediment sticks to the side of the growing branch and not just the tip region.

Similar regional trends can be observed when plotting the mean filled fraction against both stability and attraction distances (figure S3d). The same variations seen in figure S3c, where different parameter combinations lead to branching, columns, and flat layers, are captured again by this distinct measurement.

D.5 Correlation between Input Parameters and Measurements

The observed morphologies are not necessarily easily described by a few measurements, and various measurements can be used to describe different aspects of the morphology. These may correlate with each other and the input parameters in many ways. In figure S4, we plot the correlation between the input parameters and the measured quantities for both parameter sweeps to provide a concise way to summarise the interactions.

The differences in correlation between figures S4a and S4b highlight the impact that the choice of parameters to sweep can have on the observed correlation. Trends similar to those previously observed can be picked out of the correlation matrices. The stability distance controls the transition between flat layers and columns. The attraction distance controls the emergence of branching. Increasing precipitation leads to thicker layers and smooths out the structure. The correlation matrices also show the correlation between measurements, showing how different measurements can capture similar aspects of the structure.

E Additional Model Details

E.1 Initial Conditions Setup

The morphologies which emerge from the simulations are highly dependent on initial conditions. The flexibility of our framework allows initial conditions with a wide variety of structures. In this work, we use several examples. The simplest is to simply set all cells at the base of the grid to the value 1, representing a solid substrate. Another option is to localise this substrate such that the 10 cells at the centre of the base of the grid are set to solid and the rest are left empty. We also give the option for a few more complex shapes. Firstly, in order to make the structure more conical, we allow triangular initial conditions where we generate a solid triangle at the centre of the grid 100 cells tall and 30 cells wide at the base. We also allow the user to generate a small mound at the centre of the bottom of the grid, of height 10 and width 150, where the height decays quadratically. The most complex initial condition we implement is a sinusoidal function to simulate roughness. This function fills in all the cells below an integer generated by rounding $5\sin(\frac{2\pi x}{300}) + |\eta|$ where x is the distance across the base and η is a normally distributed random variable generated using the `randn()` function. Initial conditions can be modified as the user desires but we do not focus on more complex initial conditions in this work. One could, for example, modify the roughness function to make the behaviour more realistic, and the noise stronger.

E.2 Morphospace Setup

The code automatically places figures in the morphospace, automatically accounting for the size and number of figures. However, to make the output easier to read in the main text, we manually alter the spacing between plots and the background colours of the canvas on which the plots are placed. All actual simulation morphospace plots are directly output by the code.

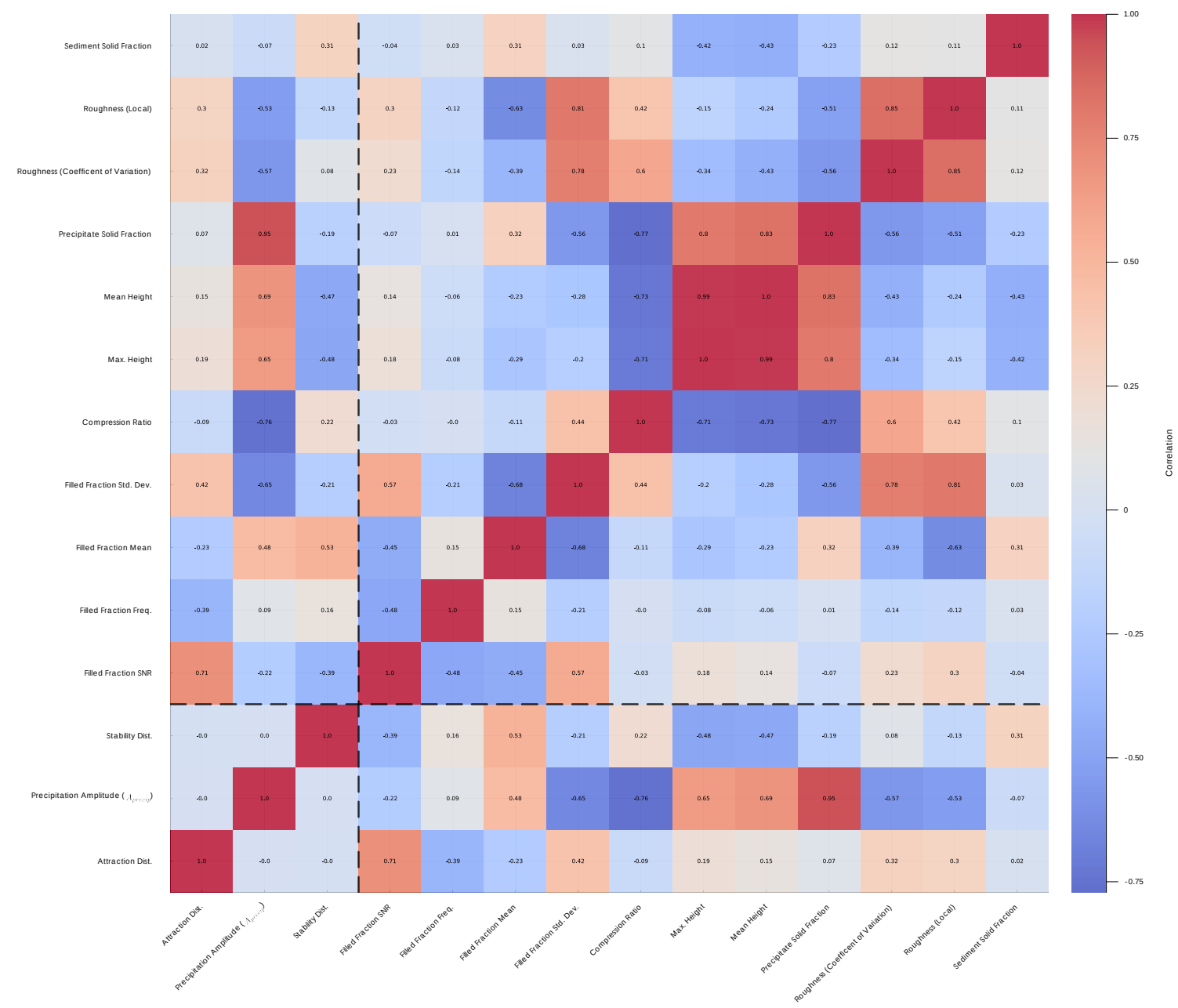

(**a**) Correlation matrix derived from varying precipitation amplitude, stability distance and attraction distance. All other parameters fixed.

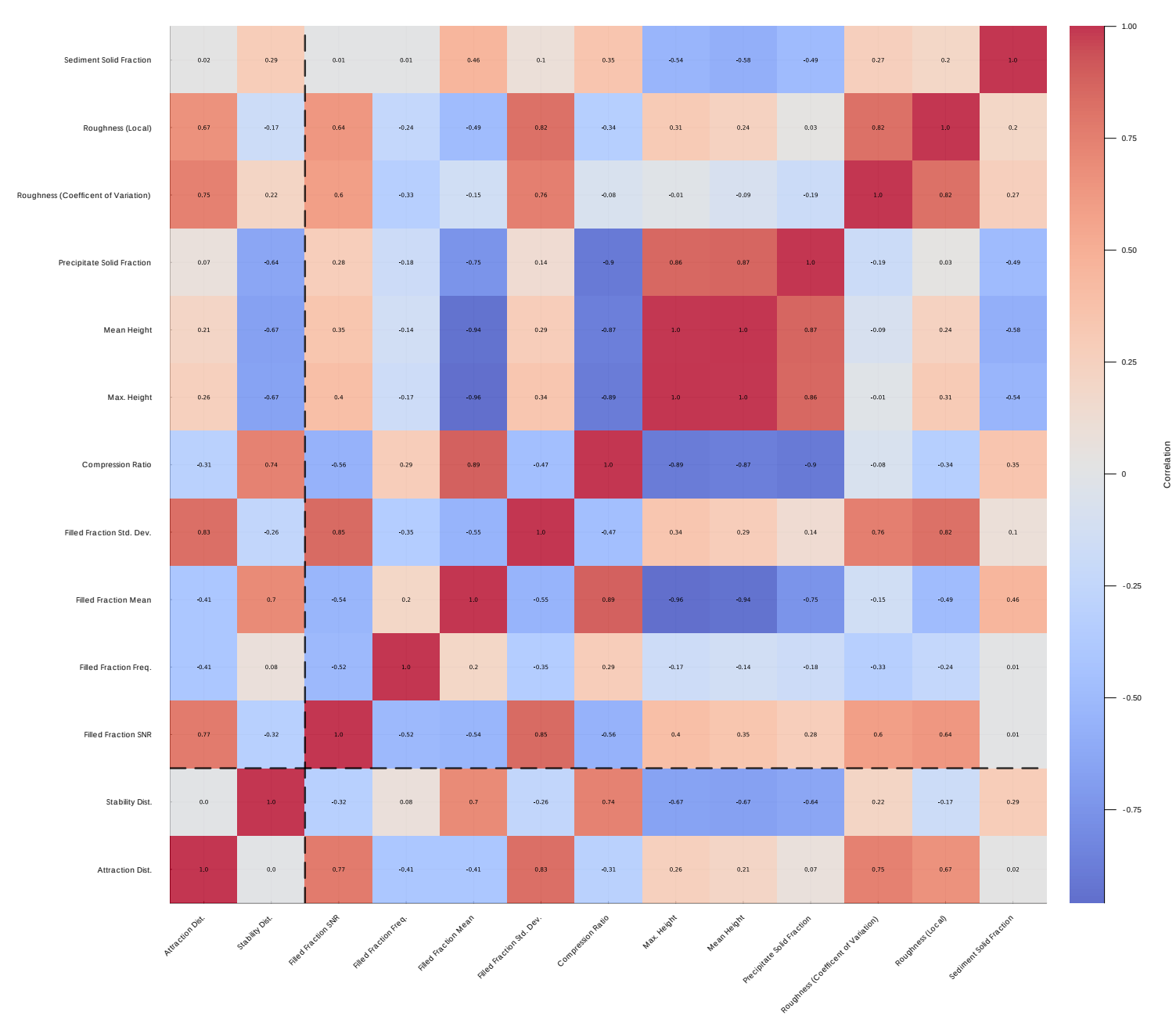

(**b**) Correlation matrix derived from varying stability distance and attraction distance. All other parameters fixed.

Figure S4. Correlation matrices obtained when fixing and sampling different regions of parameter space. Varying precipitation growth rate suppressing trends visible in the stability and attraction distance only correlation matrix. Correlation values represent the Pearson correlation coefficient.

F Parameter Values Set

In this section, we describe the set of parameters used in various scenarios. For ease of use, the code assumes a set of default parameters, and then a user only needs to input the parameters when they deviate from the default values. In this document, we list the default parameters and then highlight when we use values which deviate from the defaults given in table S1. The default parameters are not special or optimal values and should be thought of as arbitrarily selected within the range leading to functional results.

Table S1. Default Parameters Used in the Simulation

**Parameter**	**Type**	**Default**	**Description**
`width`	`Int`	1000	Width of the domain.
`height`	`Int`	400	Height of the domain.
`initial_solid_setup_type`	`Symbol`	`:base_line`	Initial condition type (`:rough`, `:mound`, `:triangle` or `:base_line`).
`downspeed`	`Int`	3	Vertical step speed for particles.
`sidespeed`	`Int`	1	Horizontal step speed for particles.
`precip_amplitude`	`Float64`	0.08	Maximum Probability of Precipitation, coefficient of precipitation periodicity function
`remove_probs`	`Float64`	0.02	Probability of precipitating agent removal per time step.
`precip_weights`	`Vector`	$[0, 1, 1]$	Growth direction preferences (down, side, up).
`sediment_amplitude`	`Int`	50	Maximum value for the sedimentation rate, coefficient of sediment periodicity function.
`period_control_T`	`Float64`	200	Sets length of oscillations for given periodic functions (proportional to period, exact realtionship depends on function choice)
`precip_period_scaling`	`Float64`	1	Relative scaling of precipitation vs. sedimentation period.
`precip_period_offset`	`Float64`	50	Phase offset for precipitation oscillations.
`stability_dist`	`Int`	15	Stability distance for the DLA process.
`attraction_dist`	`Int`	15	Attraction distance for the DLA process.
`conical_scale`	`Float64`	0	Bias for tip-growth (conical structures), set to zero for no bias.
`distance_metric`	`Symbol`	`:euclidean`	Metric used for distances (`:euclidean`, `:manhattan`, `:chebyshev`[may give non-physical behaviour]).
`initial_microbe_density`	`Float64`	0.9	Initial substrate precipitating agent proportion.
`recolonisation_prob`	`Float64`	0.5	Likelihood of precipitating agents appearing on new sediment.
`sediment_periodic_mode`	`Symbol`	`:abs`	Periodic function behaviour for sediment. Options `:abs`,`:max_cos`, `:cos2`, `:constant`
`precip_periodic_mode`	`Symbol`	`:abs`	Periodic function behaviour for microbes. Options `:abs`,`:max_cos`, `:cos2`, `:constant`
`prevent_overhang`	`Bool`	`false`	Flag to restrict horizontal overhangs.

In the following subsections, we list all the deviations from the default parameters used to generate the figures in the main text.

F.1 Parameters Used for Comparisons to Real Objects

In this section, we list the non-default parameters which were set in order to match the qualitative features of several categories of real microbialites presented in the main text. The parameters used give the desired structure; however, in general there are likely many more parameter sets that could give similar structures, which we do not fully and exhaustively explore. Additional combinations can be tested using the interactive framework.

The Goose Egg Formation structure with flat laminations relies on small stability and attraction distances, and uses the parameters given in table S2.

Table S2. Non-Default Parameters used to reproduce Goose Egg Formation flat laminations

**Parameter**	**Value**
`width`	2000
`height`	1900
`precip_amplitude`	0.05
`precip_weights`	[1,1,10]
`sediment_amplitude`	100
`period_control_T`	2000
`stability_dist`	3
`attraction_dist`	1
`recolonisation_prob`	0.99
`sediment_periodic_mode`	`:max_cos`
`precip_periodic_mode`	`:max_cos`

Domal morphologies can be formed in a similar way to planar laminations aside from using localised initial conditions, slightly more precipitation, and a more even growth axis. table S3.

More irregular layers which tend towards columnar structures can be formed by increasing the stability and attraction distances reproducing the qualitative structure of the linked-columnar stromatolite from the El Molino Formation, table S4.

Finally, branching can be induced by imposing a higher attraction distance with a low stability distance. In addition to using localised initial conditions, this allows us to reproduce the branched domal microbialite of the Howe Limestone Member listed in the main text, table S5.

F.2 Morphospace Parameters

When creating the morphospace plots we leave all parameters with their default values aside from those directly written on the figures or mentioned in the table S6 for the non-conical morphospaces and table S7 for the conical morphospaces.

F.3 Quantitative Sweep Parameters

When performing the quantitative parameter sweep we fix the non-default values shown in table S8, and sweep over the parameters in the range and density described in the quantitative analysis

Table S3. Non-Default Parameters used to reproduce Nash Fork Formation Stromatolite

Parameter	Value
width	2000
height	1900
initial_solid_setup_type	:localised
precip_amplitude	0.1
precip_weights	[1,1,3]
sediment_amplitude	100
period_control_T	2000
stability_dist	3
attraction_dist	1
recolonisation_prob	0.99
sediment_periodic_mode	:max_cos
precip_periodic_mode	:max_cos

Table S4. Non-Default Parameters used to reproduce linked-columnar stromatolite

Parameter	Value
width	2000
height	1950
precip_amplitude	0.08
precip_weights	[1,1,3]
sediment_amplitude	100
period_control_T	2000
stability_dist	35
attraction_dist	20
recolonisation_prob	0.99
sediment_periodic_mode	:max_cos
precip_periodic_mode	:max_cos

Table S5. Non-Default Parameters used to make Branched Domal Microbialite from the Howe Limestone Member formation

Parameter	Value
width	2000
height	1900
initial_solid_setup_type	:localised
precip_amplitude	0.1
precip_weights	[1,1,3]
sediment_amplitude	100
period_control_T	2000
stability_dist	5
attraction_dist	20
recolonisation_prob	0.99
sediment_periodic_mode	:max_cos
precip_periodic_mode	:max_cos

Table S6. Non-Default Parameters Used in the Non-Conical Morphospace

Parameter	Value
width	700
height	1000
precip_weights	[1,1,3]
period_control_T	1000
recolonisation_prob	0.99
sediment_periodic_mode	:max_cos
precip_periodic_mode	:max_cos

Table S7. Non-Default Fixed Parameters Used in the Conical Morphospace

**Parameter**	**Value**
`width`	700
`height`	1000
`initial_solid_setup_type`	`:triangle`
`remove_probs`	0
`precip_weights`	[0,1,10]
`sediment_amplitude`	25
`period_control_T`	1000
`conical_scale`	2
`recolonisation_prob`	0.99
`sediment_periodic_mode`	`:max_cos`
`precip_periodic_mode`	`:max_cos`
`prevent_overhang`	`true`

section in the main text. In the main text, we show a few examples of generated single stromatolites when discussing equifinality. For the first sweep, we use *sim_20260317_143341_f10c02e5* which has roughness value 0.0080, stability distance 43, attraction distance 6 and precipitation amplitude 0.2938 compared to which has *sim_20260317_143302_a96b6ea8* roughness value 0.0115, stability distance 46, attraction distance 47 and precipitation amplitude 0.2942. They have a broadly similar structure for very different parameters. While for the second sweep, we use *sim_20260317_144700_c365419c* mean filled fraction 0.9886, stability distance 35, and attraction distance 18 compared to *sim_20260317_144627_6f24fc37* mean filled fraction 0.9976, stability distance 48, and attraction distance 1. These examples have quite different parameters and structures but similar mean filled fraction.

Table S8. Non-Default Parameters Used in The Quantitative Parameter Sweeps

**Parameter**	**Value**
`width`	700
`height`	1500
`sediment_amplitude`	70
`period_control_T`	1000
`sediment_periodic_mode`	`:max_cos`
`precip_periodic_mode`	`:max_cos`

F.4 Video Parameters

We generate a few videos to highlight the model dynamics. For each video, we keep all non-default parameters the same and pick pairs of stability and attraction distances. The non-default parameters are given by table S9.

The stability and attraction distances are given in the following manner: [video 1, video 2, video

Table S9. Non-default Video Parameters (does not include stability and attraction distance values, see text)

Parameter	Value
`width`	2000
`height`	1900
`precip_amplitude`	0.1
`precip_weights`	[1,1,3]
`sediment_amplitude`	100
`period_control_T`	2000
`recolonisation_prob`	0.99
`sediment_periodic_mode`	`:max_cos`
`precip_periodic_mode`	`:max_cos`

3] = [[30,30], [5,30], [30,2]] where the order is given by [stability distance, attraction distance]. Videos are run for 2000 frames at 15 steps per frame.

G Quantities Measured

In order to capture the emergent structures quantitatively, we devise a range of simple measurements which capture various aspects of the morphology.

We measure the following parameters:

1. Sediment Fraction - Fraction of cells of the total grid filled by sediment.
2. Precipitate Fraction - Fraction of cells of the total grid filled by precipitate.
3. Scaled maximum height - Maximum height of the structure scaled by the grid height
4. Scaled mean height - Mean height of the structure per column scaled by the grid height
5. Roughness (Coefficient of Variation) - Standard Deviation of the column heights divided by the mean column height.
6. Roughness (local) - Root Mean Square (RMS) of the local height differences between columns.
7. Mean Filled Fraction (column-wise) - Mean proportion of filled cells in each column up to the maximum highest filled cell in that column
8. Standard Deviation of Filled Fraction (column-wise) - Standard Deviation of the proportion of filled cells in each column up to the maximum highest filled cell in that column
9. FFT derived frequency of Filled Fraction (column-wise) - Frequency of oscillation in filled fraction derived from FFT
10. FFT derived signal-to-noise-ratio of Filled Fraction (column-wise) - Signal-to-noise-ratio of the FFT peak derived from the filled fraction

11. Gzip Compression ratio - The size of the uncompressed file divided by the size of the compressed file; larger means more compression and regularity. We obtain this by calling `transcode(GzipCompressor, ...)` inside `Julia`.

This list of measurements is simply a subset of the many potential measurements that could be made to describe the outputs from our model. These parameters are selected based on the fact that they are computationally efficient, reasonably easy to interpret and fulfil the needs of this work. Quantification of bespoke morphologies in more detail may require a more bespoke measurement set.

References

[1] A. P. Petroff et al. "Biofilm Growth and Fossil Form". In: *Physical Review X* 3.4 (Nov. 2013), p. 041012. ISSN: 2160-3308. DOI: 10.1103/PhysRevX.3.041012.

[2] P. Szymczak et al. "Shapes of ideal stalagmites". In: *Proceedings of the National Academy of Sciences of the United States of America* 122.42 (2025). ISSN: 10916490. DOI: 10.1073/pnas.2513263122.

[3] C. Dupraz, R. Pattisina, and E. Verrecchia. "Translation of energy into morphology: Simulation of stromatolite morphospace using a stochastic model". In: *Sedimentary Geology* 185.3-4 (Mar. 2006), pp. 185–203. ISSN: 00370738. DOI: 10.1016/j.sedgeo.2005.12.012.

[4] F. A. Corsetti and M. C. Storrie-Lombardi. "Lossless Compression of Stromatolite Images: A Biogenicity Index?" In: *Astrobiology* 3.4 (Dec. 2003), pp. 649–655. ISSN: 1531-1074. DOI: 10.1089/153110703322735980.

[5] K. L. Wagstaff and F. A. Corsetti. "An Evaluation of Information-Theoretic Methods for Detecting Structural Microbial Biosignatures". In: *Astrobiology* 10.4 (May 2010), pp. 363–379. ISSN: 1531-1074. DOI: 10.1089/ast.2008.0301.
